# Direct and Regularized Inverse De-Embedding for Single-Carrier Signal Recovery in Measurement Front-Ends

**DOI:** 10.3390/s26123629

**Published:** 2026-06-06

**Authors:** Haonan Gu, Yingxin Jin, Yongnan Rao, Decai Zou, Yongpeng Liu

**Affiliations:** 1 National Time Service Center, Chinese Academy of Sciences, Xi’an 710600, China; guhaonan22@mails.ucas.ac.cn (H.G.);; 2 Key Laboratory of Precision Navigation and Timing Technology, Chinese Academy of Sciences, Xi’an 710600, China; 3 University of Chinese Academy of Sciences, Beijing 100049, China

**Keywords:** single-carrier signal, de-embedding, measurement front-end, ill-posed inverse problem, regularized inverse compensation, Wiener inverse filtering

## Abstract

**Highlights:**

**What are the main findings?**

- A frequency-domain de-embedding compensation framework is established for single-carrier signal recovery in measurement chains, and the instability of direct inverse compensation under weak-response regions and observation noise is analyzed.
- Direct, Tikhonov, Wiener-type, and Truncated methods are compared under different measurement-chain and SNR conditions. Wiener-type inverse compensation achieves better NMSE performance, while Tikhonov and Truncated methods mainly improve stability by limiting excessive inverse gain.

**What are the implications of the main findings?**

- The proposed framework provides a practical approach for $S_{21}$-based signal-level recovery of measured single-carrier signals, rather than simple response correction.
- The results clarify method applicability: Direct compensation is suitable for relatively flat measurement-chain responses, whereas regularized inverse compensation is more valuable under low-SNR conditions or local weak-response regions.

**Abstract:**

To address the degradation of recovery accuracy caused by amplitude fluctuation, phase distortion, delay distortion, and noise amplification in single-carrier signal measurement chains, this paper investigates direct inverse and regularized inverse de-embedding compensation methods. Based on a linear time-invariant system model, single-carrier signal de-embedding is formulated as an ill-conditioned inverse problem that is sensitive to weak-response frequency points and observation noise. A unified frequency-domain compensation framework is then established, including the Direct method, Tikhonov regularized inverse compensation, Wiener-type inverse compensation, and truncated inverse compensation. To evaluate the applicability of these methods, a narrowband single-carrier signal and four measurement-chain models are constructed, including a smooth reference chain, a passband-edge attenuation chain, a multiple local-fading ill-conditioned chain, and a measured S-parameter-based chain. The simulation results show that the compensation gain is closely related to the magnitude response of the measurement chain. The Direct, Tikhonov, and Truncated methods produce similar results when the chain response is relatively flat or when the regularization constraint is weak, whereas the Wiener-type method achieves better NMSE performance under the tested conditions. Parameter-sweep and SNR experiments further show that the effectiveness of regularized inverse compensation depends on the ill-conditioning degree of the measurement chain, the noise level, and the parameter settings. Measured single-carrier signal experiments verify the feasibility of the proposed framework. Frequency-domain de-embedding compensation based on the measured $S_{21}$ improves the NMSE from −18.7808 dB before compensation to −37.9458 dB after compensation. The measured results also show that, when the measurement-chain response is relatively flat, the additional improvement of Tikhonov and Truncated methods over the Direct method is limited, while the Wiener-type method provides a slight NMSE improvement. Overall, the proposed framework provides a practical approach for single-carrier signal recovery and clarifies the applicability of different inverse compensation methods under different measurement-chain and noise conditions.

## 1. Introduction

In RF testing, signal measurement, and system characterization, components in the measurement chain, such as cables, connectors, filters, amplifiers, and sampling front ends, introduce amplitude fluctuations, phase distortion, and delay distortion into the signal under test, causing the acquired data to deviate from the true spectral and temporal characteristics of the original signal [1,2]. This issue is particularly critical in high-precision measurement scenarios, where the additional influence of the measurement chain cannot be neglected. Without proper correction, such distortions directly limit the accuracy of subsequent parameter estimation, performance evaluation, and distortion analysis. Therefore, de-embedding compensation, as a key technique for removing the influence of the measurement chain and recovering the intrinsic characteristics of the signal, has become an important research topic in precision measurement and signal recovery [3].

The basic principle of de-embedding is to compensate for the amplitude and phase distortions introduced into the observed signal by the measurement chain, using known or estimable transmission characteristics of the measurement chain, so as to recover the original signal at the input of the measurement chain as accurately as possible [3]. In general, the measurement chain can be characterized by its frequency response H(f), which essentially represents the combined amplitude and phase effects imposed on the input signal. For a two-port network, the forward transmission parameter $S_{21}(f)$ is commonly used as a direct representation of the transmission characteristics of the measurement chain [3].

In the field of RF and microwave measurements, S-parameter-based fixture de-embedding and reference-plane calibration have developed into relatively mature technical frameworks [4]. Typical methods include TRL and multiline TRL calibration [5,6,7], asymmetric fixture de-embedding [8], and automatic fixture removal methods [7,9]. These methods are mainly intended for device or network-parameter measurements. Their objective is to establish an equivalent error model between the measurement plane and the device reference plane, and then remove the effects of fixtures, cables, and interconnection structures through matrix operations [8,9,10,11,12,13].

However, for practically acquired single-carrier signals, the amplitude–frequency fluctuation, phase delay, and noise perturbation of the measurement chain directly affect the signal spectrum and time-domain waveform. Therefore, the de-embedding problem involves not only the acquisition of the measurement-chain response, but also noise amplification and signal recovery stability during inverse compensation. Accordingly, de-embedding compensation for single-carrier signal recovery should further account for measurement-chain response uncertainty and compensation stability.

In existing studies, the measurement-chain response H(f) used for compensation is usually obtained in two ways. The first approach constructs the measurement-chain response from S-parameters measured by a vector network analyzer. Specifically, the amplitude and phase transmission characteristics of the device or measurement chain are obtained through offline measurement of $S_{21}(f)$ [3]. This approach has a clear physical interpretation and can provide relatively complete amplitude and phase information of the measurement chain. However, the measured results mainly reflect the static characteristics under offline calibration conditions, which may differ from the actual measurement-chain response during signal acquisition.

The second approach estimates the measurement-chain response from measured input and output data. When both the reference branch and the measurement branch are available, the frequency response can be estimated using the ratio of the cross-power spectral density between the two signals to the auto-power spectral density of the reference-branch signal [14]. This approach can more directly reflect the measurement-chain characteristics under actual measurement conditions. Nevertheless, its estimation accuracy is constrained by signal bandwidth, excitation strength, and noise level. Under low-SNR conditions or in weakly excited frequency regions, the response estimate may become unstable.

Therefore, whether the measurement-chain response is constructed from S-parameters or estimated from input–output data, response errors and observation noise may be further amplified during inverse compensation. This is an important reason why stabilization processing is required in de-embedding compensation for single-carrier signals.

## 2. De-Embedding Compensation Model and Regularized Inverse Compensation Method

### 2.1. De-Embedding Compensation Model

To analyze the distortion imposed by the measurement chain on a single-carrier signal, the chain can be modeled as a linear time-invariant system. Let x(t) denote the original signal at the input of the chain and y(t) denote the observed signal at the output. Then, the relationship between them can be expressed as

(1) y(t)=h(t)∗x(t)+n(t)

where h(t) is the impulse response of the measurement chain, ∗ denotes the convolution operation, and n(t) represents the observation noise and unmodeled residuals. By transforming the above expression into the frequency domain, one obtains

(2) Y(f)=H(f)X(f)+N(f)

where X(f), Y(f), and N(f) denote the frequency-domain representations of x(t), y(t), and n(t), respectively, and H(f) is the overall frequency response of the measurement chain, which characterizes the combined effect of the chain on the amplitude and phase of the input signal.

The objective of de-embedding compensation is to recover, as accurately as possible, the original input signal X(f) from the observed signal Y(f) and the chain response H(f). Under ideal conditions, if the chain response is known and no zero exists in the frequency domain, direct inverse compensation can be expressed as

(3) $$X(f)=\frac{Y(f)}{H(f)}$$

where X(f) is the recovered signal spectrum. This expression indicates that de-embedding compensation is essentially an inverse-system solution for the measurement chain. However, in practical measurements, the chain response is usually available only in an estimated form. Let $\hat{H}(f)$ denote the chain response used for compensation in practice. Then, the result of direct de-embedding can be expressed as

(4) $$\hat{X}(f)=\frac{Y(f)}{\hat{H}(f)}$$

In practical de-embedding, the recovery accuracy is jointly affected by observation noise and chain-response estimation errors. In particular, when ∣H(f)∣ becomes small at certain frequency points, direct inverse compensation produces a large inverse gain, which significantly amplifies both the noise term and the response estimation error, thereby leading to increased fluctuations, local distortion, or even overall instability in the recovered result [15]. Therefore, direct inverse compensation is inherently unsuitable for de-embedding scenarios involving small-response frequency points and noise perturbations. To address this issue, the next subsection introduces a regularized inverse compensation method, in which the inverse gain is constrained to improve the stability of the de-embedding process.

### 2.2. Regularized Inverse Compensation Method

To suppress the noise amplification caused by small-response frequency points in the de-embedding of the single-carrier signal considered in this study, the inverse compensation process must be stabilized [16,17]. For the scenario investigated herein, the measurement chain can be approximately modeled as a single-input single-output linear time-invariant system, and its frequency-domain representation is given by Equation (2). To introduce additional constraints into the inversion process and suppress the rapid amplification of noise and chain-response errors, exact inverse compensation based directly on 1/H(f) is generally no longer suitable. Instead, the inverse gain at small-response frequency points should be constrained, such that the compensation operator is transformed from an exact inverse into a stable approximate inverse. On the one hand, the spectral energy of a single-carrier signal is mainly concentrated around the carrier frequency, and some frequency regions are insufficiently excited, making the estimation of the chain response more susceptible to noise perturbations. On the other hand, when ∣H(f)∣ becomes locally small, direct inverse compensation significantly enhances the noise and error components at these frequency points, thereby increasing fluctuations in the compensation result and even degrading the overall recovery performance. Therefore, it is necessary to introduce regularization constraints into the direct inverse compensation framework to improve the stability of de-embedding under small-response frequency points and low carrier-to-noise ratio conditions. To meet this requirement, this study further considers three stable inverse compensation methods suitable for this scenario, namely, Tikhonov regularized inverse filtering, Wiener-type inverse filtering, and truncated inverse compensation [18].

#### 2.2.1. Tikhonov Regularized Inverse Filtering

For unstable solutions to ill-conditioned linear inverse problems, Tikhonov [15] proposed a stable approximate inversion method by introducing a quadratic penalty term into the least-squares formulation. Subsequent studies have shown that this method can effectively suppress error amplification along the small-singular-value directions of ill-conditioned systems and has therefore become a classical regularization approach for solving ill-posed inverse problems [15,19,20,21,22,23]. In a review on the regularization of ill-conditioned and singular linear systems, Neumaier [20] pointed out that conventional Tikhonov regularization can be derived as a modification of the standard least-squares formulation, and its typical solution can be written as

(5) $$\hat{x}={\left(A*A+h^{2}I\right)}^{-1}A*y$$

where A is the system matrix, y is the observation vector, $\hat{x}$ is the recovered result, I is the identity matrix, and $h^{2}$ is the regularization parameter. The physical implication of this expression is that, when the system matrix A is ill-conditioned, the direct least-squares inverse solution is strongly affected by small singular values. By adding the regularization term $h^{2}I$ to $A^{*}A$, the stability of the inverse solution can be improved and excessive amplification of the inverse gain can be avoided. For the scenario considered in this study, a Tikhonov regularization constraint [15] is introduced into the direct inverse compensation framework, and the de-embedding problem can therefore be reformulated as

(6) $$\underset{X(f)}{\min}\left({|Y(f)-H(f)X(f)|}^{2}+{\lambda |X(f)|}^{2}\right)$$

where λ is the regularization parameter used to balance data fidelity and solution stability. By minimizing the above expression with respect to X(f), one obtains

(7) $$\frac{\partial }{\partial X^{*}(f)}\left(|Y(f)-H(f)X(f){|}^{2}+\lambda |X(f){|}^{2}\right)=0$$

After further simplification, the Tikhonov regularized inverse compensation expression for the scenario considered in this study can be obtained as

(8) $${\hat{X}}_{reg}(f)=\frac{H^{*}(f)}{{|H(f)|}^{2}+\lambda }Y(f)$$

where $H^{*}(f)$ is the complex conjugate of the chain frequency response H(f), ${|H(f)|}^{2}$ denotes the power gain at the corresponding frequency point, and λ is the regularization parameter. Equation (15) is the frequency-by-frequency form of the Tikhonov regularized solution ${\left(A^{*}A+\lambda I\right)}^{-1}A^{*}y$ for a linear time-invariant system in the frequency domain. Compared with direct inverse compensation, the resulting Tikhonov regularized inverse compensation differs only in that an additional stabilization term, λ, is introduced into the denominator; however, this modification plays a critical role. When ${|H(f)|}^{2}\gg \lambda$, the above expression approximately reduces to direct inverse compensation. In contrast, when ${|H(f)|}^{2}$ becomes small, λ effectively limits the continuous increase in inverse gain, thereby suppressing the abnormal amplification of noise and errors at small-response frequency points.

#### 2.2.2. Wiener-Type Inverse Filtering

In addition to Tikhonov regularization, Wiener-type inverse filtering is also a commonly used stable inverse compensation method for ill-posed inverse problems [20]. The theoretical foundation of Wiener-type inverse filtering originates from Wiener’s study of optimal linear estimation for stationary random processes, with the core idea of constructing an optimal filter under the minimum mean square error criterion [24]. This method was later widely applied to deconvolution [25], denoising [26], and inverse system reconstruction [15,27,28,29,30], and has become part of the standard derivation framework in digital signal processing textbooks.

For the single-carrier signal de-embedding problem considered in this study, the frequency-domain model of the measurement chain is given by Equation (2). The objective of Wiener-type inverse filtering is to minimize the error between the recovered result $\hat{X}(f)$ and the original signal X(f) in the mean square error sense, that is, to solve

(9) $$min\;E\left\{{|X(f)-\hat{X}(f)|}^{2}\right\}$$

By substituting Equation (2) into the above expression and assuming that X(f) and N(f) are uncorrelated, the mean square error can be expressed as

(10) $$E\left\{{|X(f)-\hat{X}(f)|}^{2}\right\}={|1-W(f)H(f)|}^{2}S_{x}(f)+|W(f){|}^{2}S_{n}(f)$$

where $S_{x}(f)$ and $S_{n}(f)$ are the power spectral densities of the signal and noise, respectively. By taking the partial derivative of the above expression with respect to $W^{*}(f)$ and setting it to zero, the Wiener-type inverse compensation coefficient can be obtained as

(11) $$W(f)=\frac{H^{*}(f)S_{x}(f)}{|H(f){|}^{2}S_{x}(f)+S_{n}(f)}$$

Therefore, the Wiener-type inverse compensation expression for the scenario considered in this study can be written as

(12) $${\hat{X}}_{W}(f)=\frac{H^{*}(f)S_{x}(f)}{|H(f){|}^{2}S_{x}(f)+S_{n}(f)}Y(f)$$

It can be further rewritten as

(13) $${\hat{X}}_{W}(f)=\frac{H^{*}(f)}{|H(f){|}^{2}+\frac{S_{n}(f)}{S_{x}(f)}}Y(f)$$

where $H^{*}(f)$ is the complex conjugate of H(f), ${|H(f)|}^{2}$ denotes the power gain of the chain at the corresponding frequency point, and $\frac{S_{n}(f)}{S_{x}(f)}$ reflects the relative strength of the noise with respect to the signal. Compared with direct inverse compensation, Wiener-type inverse filtering introduces a frequency-dependent statistical stabilization term into the denominator, which enables the inverse compensation gain to be adaptively adjusted according to the signal and noise levels, thereby reducing the noise amplification effect at small-response frequency points.

#### 2.2.3. Truncated Inverse Compensation Method

In addition to regularized filtering, truncated inverse compensation is also a commonly used stabilization strategy for ill-posed inverse problems. Its theoretical basis originates from the idea of truncated singular value decomposition regularization [31], in which unstable inversion directions associated with small singular values are discarded or constrained to suppress error amplification. In inverse filtering problems, when the system frequency response approaches zero at certain frequency points, direct inverse compensation can significantly amplify measurement noise. Therefore, threshold truncation or gain limiting is often introduced to prevent the inverse gain from increasing without bound. For the single-carrier signal de-embedding problem considered in this study, the action of the measurement chain in the frequency domain is represented by pointwise multiplication. Accordingly, the small-singular-value directions in this scenario can be approximately associated with frequency points at which ∣H(f)∣ is small. In other words, when the chain-response magnitude becomes excessively small and the inverse compensation gain becomes excessively large at certain frequency points, these points constitute unstable compensation regions and must be subject to additional constraints.

Based on the above idea, this study constructs a truncated inverse compensation expression by imposing an amplitude threshold on small-response frequency points. In essence, this formulation can be regarded as an engineering implementation of the TSVD concept for a diagonal linear time-invariant system in the frequency domain [32,33,34,35]. For the scenario considered in this study, a threshold τ can be introduced, and the truncated inverse compensation expression can be written as

(14) $$\hat{X_{tr}}\left(f\right)=\{\begin{array}{c} \frac{Y(f)}{H(f)},\;\;\left|H(f)\right|\geq \tau \\ \frac{Y(f)}{\tau e^{j∠H(f)}},\;\;\left|H(f)\right|<\tau \end{array}$$

where τ is the truncation threshold, and ∠H(f) denotes the phase of the chain frequency response. This expression indicates that, when ∣H(f)∣ is greater than the threshold, the compensation result is identical to that of direct inversion. When ∣H(f)∣ is smaller than the threshold, the phase compensation remains unchanged, while the amplitude compensation gain is limited to 1/τ, thereby suppressing the continued increase in inverse gain. In this formulation, the choice of the threshold τ directly affects the performance of truncated inverse compensation. If τ is set too small, the method degenerates into a form close to direct inverse compensation and cannot effectively suppress noise amplification at small-response frequency points. In contrast, if τ is set too large, although the inverse gain can be more strongly constrained, the compensation capability at some frequency points will also be weakened, which may lead to a certain degree of under-compensation in the recovered result.

Considering that the amplitude-response ranges differ among different measurement-chain models, this study does not use a single fixed threshold τ. Instead, a relative truncation threshold is set according to the amplitude-response characteristics of each measurement chain within the effective signal bandwidth, namely:

(15) $$\tau =c\cdot \underset{f\in B}{\min}\;H_{low}\left(f\right)$$

where B denotes the effective signal bandwidth, and c is the relative truncation-strength coefficient. By varying c, different truncation states can be characterized, including inactive truncation, critical activation, and strong truncation. When c < 1, τ is smaller than the minimum in-band magnitude of the measurement-chain response, and the truncation constraint is generally not activated. In this case, the method approaches direct inverse compensation. When c ≈ 1, the truncation constraint is first activated near the minimum-response frequency point. When c > 1, the truncated frequency range gradually expands, which can more strongly limit the inverse gain at small-response frequency points but may also lead to under-compensation.

### 2.3. Evaluation Metrics

To quantitatively evaluate the recovery performance of different inverse compensation methods for single-carrier signals, this study adopts the normalized mean square error and the amplitude error as evaluation metrics. These two metrics are used to analyze the compensation results from two aspects, namely, the overall reconstruction accuracy and the fidelity of the spectral amplitude.

#### 2.3.1. Normalized Mean Square Error

The normalized mean square error is used to quantify the overall deviation between the recovered signal after compensation and the original signal, and is defined as [36]

(16) $$NMSE=10{log}_{10}\frac{\sum_{n}{|x(n)-\hat{x}(n)|}^{2}}{\sum_{n}{|x(n)|}^{2}}$$

where x(n) denotes the original signal at the input of the measurement chain, and $\hat{x}(n)$ denotes the recovered signal obtained after inverse compensation. This metric is used to measure the overall energy error of the recovered signal relative to the original signal and is normalized by the energy of the original signal to reduce the influence of different signal amplitude levels on the error evaluation. For convenience of comparison, the NMSE is expressed in dB in this study. A smaller NMSE indicates that the compensated signal is closer to the original signal and that the overall recovery accuracy is higher.

#### 2.3.2. Amplitude Error

In addition to the overall reconstruction accuracy, de-embedding compensation should also consider the ability to preserve the spectral amplitude of the recovered signal in the frequency domain. Therefore, this study further introduces the amplitude error metric to quantify the average deviation in spectral magnitude between the recovered signal and the original signal. It is defined as

(17) $$AmpError=\frac{1}{N}\sum_{k=1}^{N}|20{log}_{10}\left(|X(k)|+\varepsilon \right)-20{log}_{10}\left(|\hat{X}(k)|+\varepsilon \right)|$$

where X(k) and $\hat{X}\left(k\right)$ denote the spectra of the original signal and the recovered signal, respectively. ε is a numerical protection term introduced to avoid taking the logarithm of zero. In all experiments in this study, ε is fixed at $10^{-12}$. This parameter is used only for the calculation of AmpError and is not involved in the de-embedding compensation process. AmpError is expressed in dB and is used to characterize the average spectral-amplitude distortion of the compensated signal in the frequency domain. A smaller AmpError indicates that the spectral magnitude of the recovered signal is closer to that of the original signal, corresponding to better frequency-domain amplitude fidelity.

## 3. Simulation Measurement-Chain Design and Experimental Parameter Settings

### 3.1. Construction of the Simulated Signal

To verify the effectiveness of the above compensation methods in a single-carrier signal scenario, a narrowband single-carrier simulated signal was constructed as the test input. The main parameters are listed in Table 1. The simulated signal was generated using a three-step procedure, namely, complex baseband generation, frequency-domain band limitation, and up-conversion. The generation process is described as follows.

First, an independently and identically distributed Gaussian random sequence was generated in the complex baseband domain to construct the initial complex baseband signal. To impose a finite bandwidth on the signal, frequency-domain band-limiting was then applied, and only the spectral components within ±2 MHz around zero frequency were retained, thereby obtaining a narrowband complex baseband signal with a bandwidth of 4 MHz. This process can be expressed as

(18) $$B(f)=B_{0}(f)\cdot H_{\mathrm{bb}}(f)$$

where $B_{0}(f)$ is the spectrum of the initial complex Gaussian random sequence, and $H_{bb}(f)$ is the baseband band-limiting function. In this study, $H_{bb}(f)$ is selected as an ideal low-pass function in the simulation. After this processing, the resulting signal is no longer a full-band random process, but a narrowband complex baseband signal whose spectral energy is concentrated within a limited range around zero frequency. The obtained complex baseband signal is then digitally up-converted to the vicinity of the carrier frequency, yielding a real-valued sampled carrier signal:

(19) $$x(t)=R\{b(t)e^{j2\pi f_{c}t}\}$$

where b(t) is the band-limited complex baseband signal, $f_{c}$ is the carrier frequency, and R{⋅} denotes the real-part operation. After this step, the signal spectrum is shifted from baseband to around 180 MHz, forming a narrowband spectral structure symmetrically distributed on both sides of $f_{c}$. To ensure comparability under different experimental conditions, amplitude normalization is further applied to the generated signal so that its mean-square value remains consistent.

**Figure 1:**
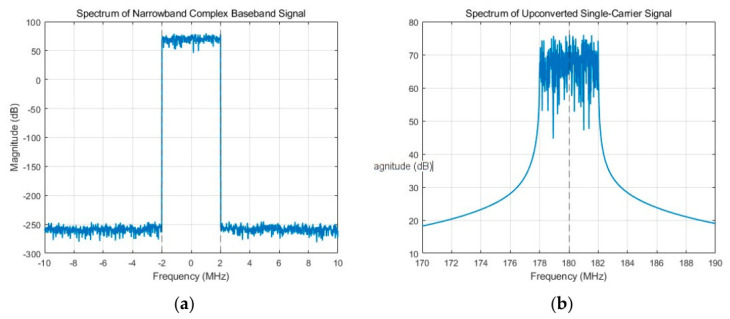
Spectra of the Simulated Signals. (**a**) Spectrum of the Narrowband Complex Baseband Signal. (**b**) Spectrum of the Up-Converted Single-Carrier Signal.

Figure 1a shows the spectrum of the narrowband complex baseband signal. It can be seen that, after frequency-domain band limitation, the signal energy is mainly concentrated within approximately ±2 MHz around zero frequency. The in-band magnitude remains relatively flat, while the out-of-band components rapidly decrease to the vicinity of the noise floor, indicating that the constructed complex baseband signal exhibits good bandwidth-constrained characteristics. Figure 1b shows the spectrum of the up-converted single-carrier signal. It can be observed that the baseband spectrum is shifted to around 180 MHz and is symmetrically distributed on both sides of the carrier frequency, forming a typical narrowband single-carrier spectral structure. These results demonstrate that the constructed signal preserves the narrowband characteristics of a single-carrier signal while maintaining a finite spectral width, making it suitable for analyzing the effect of the chain frequency response on de-embedding compensation performance.

### 3.2. Simulation Chain Model Construction

To compare the recovery capability of different inverse compensation methods under different measurement-chain conditions, this study further constructs several types of test measurement chains based on the ideal narrowband single-carrier signal. According to the de-embedding compensation model established in Section 2, the effect of the noiseless test measurement chain in the frequency domain can be expressed as:

(20) $$Y_{0}\left(f\right)=H\left(f\right)X\left(f\right)$$

where X(f) denotes the spectrum of the input signal, H(f) denotes the frequency response of the measurement chain, and $Y_{0}\left(f\right)$ denotes the output spectrum of the measurement chain under noiseless conditions. In the simulation, H(f) is used to characterize the combined amplitude and phase effects imposed by the measurement chain on the input signal, and consists of the magnitude response and the phase response.

In the subsequent de-embedding compensation process, the measurement-chain response used for compensation is denoted as $\hat{H}\left(f\right)$. For artificially constructed simulation measurement chains, $\hat{H}\left(f\right)$ is directly given by the corresponding chain model. For the measurement-chain model based on measured S-parameters, $\hat{H}\left(f\right)$ is obtained by interpolating the measured $S_{21}$ data onto the simulation frequency grid.

This study focuses on comparing the de-embedding compensation performance of different inverse compensation forms under given measurement-chain response conditions. Therefore, this section mainly describes the construction of the measurement-chain response models. Considering that different response shapes may affect inverse compensation stability in different ways, four measurement-chain models are constructed, including a smooth reference response, a passband-edge attenuation response, a multiple local-fading response, and a measured S-parameter response. These models are used to evaluate the applicability and performance differences of different inverse compensation methods under conditions ranging from weak distortion to pronounced ill-conditioning.

#### 3.2.1. Smooth Reference Chain

The smooth reference chain is used to characterize the typical response of a measurement chain under non-ill-conditioned conditions and serves as the baseline model for the subsequent analysis of various ill-conditioned chains. In this model, the amplitude response remains smooth and continuous over the entire frequency range, without introducing significant local fading or deep notches. Only a small passband fluctuation is imposed in the vicinity of the carrier frequency to represent the mild amplitude nonuniformity commonly observed in practical devices. Meanwhile, a linear delay term and a small nonlinear phase perturbation are introduced into the phase response to characterize the propagation delay and phase fluctuation generally present in measurement chains. This chain model can be used to analyze the basic recovery performance and differences between direct inverse compensation and various regularized inverse compensation methods under relatively ideal conditions.

**Figure 2:**
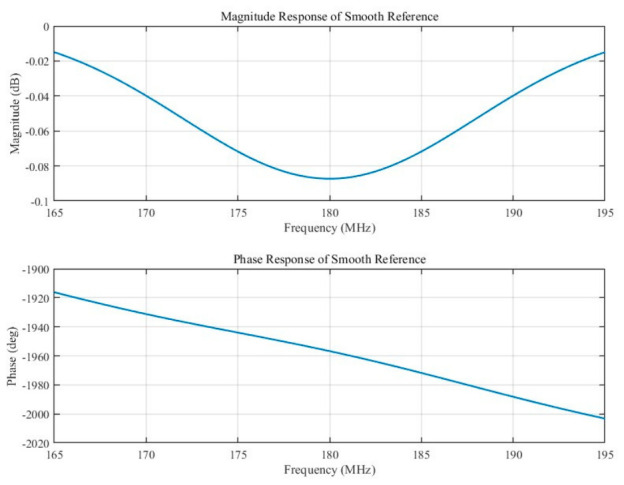
Magnitude and Phase Responses of the Smooth Reference Chain.

As shown in the Figure 2, the amplitude response of the smooth reference chain varies gradually over the frequency range from 165 MHz to 195 MHz, without exhibiting obvious local depressions, deep notches, or abrupt fading. Only a slight and continuous dip appears in the vicinity of the carrier frequency at 180 MHz, and the overall amplitude fluctuation remains small, at approximately the 0.07 dB level. The phase response shows a smooth and monotonic variation with increasing frequency and generally exhibits an approximately linear decreasing trend. Only a small and slowly varying perturbation is superimposed on the phase curve, while no evident phase discontinuity or severe distortion is observed.

#### 3.2.2. Passband-Edge Attenuation Chain

The passband-edge attenuation chain is used to simulate the response characteristic of practical filters or analog front ends, in which the amplitude gradually rolls off near the edges of the effective bandwidth. Unlike the smooth reference chain, the amplitude response of this model is no longer approximately constant over the entire passband. Instead, it gradually decreases as the frequency moves away from the carrier frequency, thereby forming a continuous amplitude roll-off region near the edges of the effective signal bandwidth. Although this type of chain does not exhibit abrupt deep notches, it already shows weak-response characteristics in the passband-edge region, causing the inverse compensation gain to increase progressively and the noise amplification effect to become more pronounced. Based on this model, the recovery capability of different inverse compensation methods under progressively weakened response conditions can be further analyzed, and their abilities to suppress passband-edge distortion can be compared.

**Figure 3:**
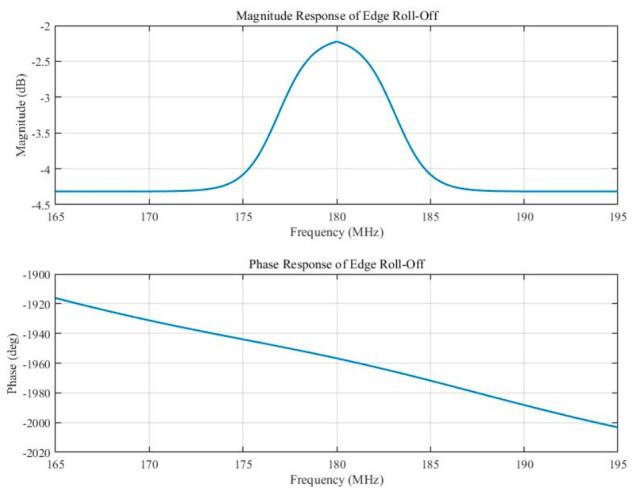
Magnitude and Phase Responses of the Passband-Edge Attenuation Chain.

As shown in the Figure 3, the amplitude response of the passband-edge attenuation chain exhibits a clear distribution with a higher response near the center and lower responses toward the band edges over the frequency range from 165 MHz to 195 MHz. In the vicinity of the carrier frequency at 180 MHz, the amplitude response is approximately −2.2 dB. As the frequency moves toward both band edges, the response gradually decreases and reaches approximately −4.3 dB near 165 MHz and 195 MHz, corresponding to an additional edge attenuation of about 2.1 dB relative to the center frequency. In terms of the phase response, the chain shows an overall smooth and monotonic decreasing trend over the range from 165 MHz to 195 MHz, without any obvious phase discontinuity or severe bending.

#### 3.2.3. Multiple Local-Fading Ill-Conditioned Chain

The multiple local-fading ill-conditioned chain is a further extension of the single local-notch model and is used to simulate practical complex chains in which multiple weak-response frequency points may exist simultaneously. Unlike the single local-notch model, which contains only one ill-conditioned region, this model introduces local fading at multiple frequency locations around the carrier frequency, causing the chain frequency response to exhibit small-amplitude characteristics in several frequency regions simultaneously. Under this condition, the inverse compensation process is no longer dominated by a single ill-conditioned point, but is jointly affected by multiple locally unstable regions, and therefore more closely resembles the frequency-response characteristics of practical complex measurement chains.

**Figure 4:**
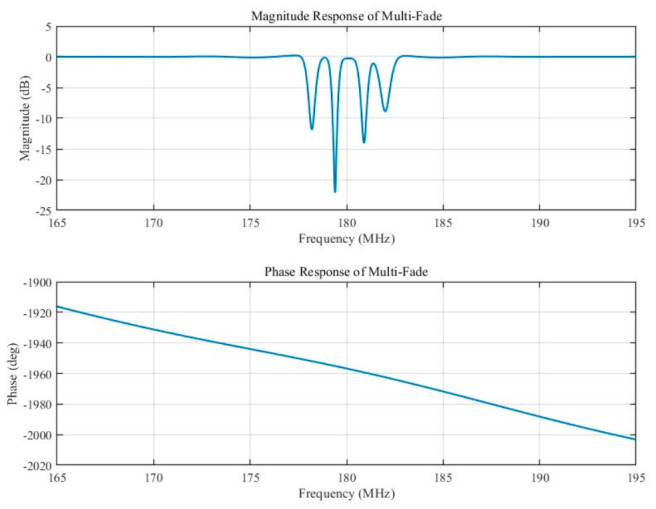
Magnitude and Phase Responses of the Multiple Local-Fading Ill-Conditioned Chain.

As shown in the Figure 4, the multiple local-fading ill-conditioned chain exhibits pronounced multi-point weak-response characteristics over the frequency range from 165 MHz to 195 MHz. Specifically, four local fading points are present within approximately 178 MHz to 182 MHz around the carrier frequency. The deepest fading occurs at approximately 179.4 MHz, where the magnitude drops to about −22 dB. The other fading points are located near 178.2 MHz, 180.9 MHz, and 181.9 MHz, with minimum magnitudes of approximately −12 dB, −14 dB, and −9 dB, respectively. Outside these local fading regions, the magnitude response remains close to 0 dB, with only minor fluctuations. In terms of the phase response, the chain exhibits an overall smooth and monotonically decreasing trend over the range from 165 MHz to 195 MHz. The phase curve is approximately linear as a whole, without any obvious discontinuities.

#### 3.2.4. Chain Model Based on Measured S-Parameters

To enhance the engineering realism of the simulation analysis, a chain model based on measured S-parameters is further introduced in this study. Specifically, the $S_{21}(f)$ data of the filter or measurement chain are read and used as the chain frequency response H(f) in the simulation, thereby establishing a frequency-domain model consistent with the characteristics of the actual device. Compared with artificially constructed models, such as the smooth response, passband-edge attenuation, or local-notch models, the chain model based on measured S-parameters can more realistically reflect the combined characteristics of practical chains, including amplitude fluctuations, passband-edge roll-off, and local weak-response regions.

**Figure 5:**
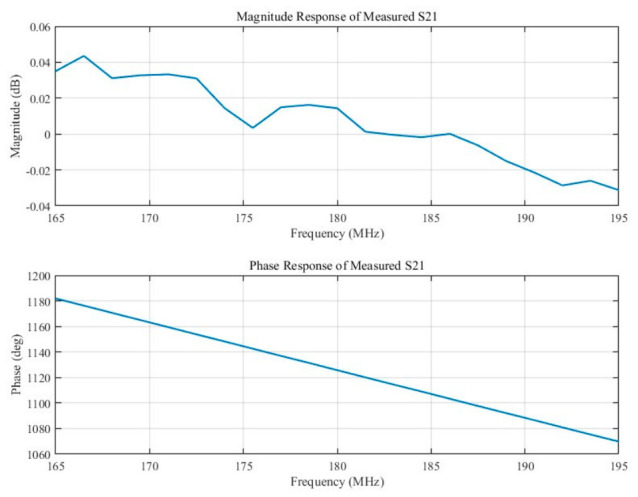
Magnitude and Phase Responses of the Measured $S_{21}$ Chain.

As shown in the Figure 5, the chain model based on the measured $S_{21}$ exhibits an amplitude response characterized by slow fluctuations superimposed on a mild decreasing trend over the frequency range from 165 MHz to 195 MHz. Specifically, the magnitude is approximately 0.035 dB near 165 MHz, followed by slight fluctuations in the range of 166 MHz to 168 MHz, where the local maximum reaches approximately 0.043 dB. Thereafter, the amplitude response gradually decreases and drops to about 0.004 dB near 175 MHz. A small local recovery is then observed in the range of 176 MHz to 180 MHz, where the magnitude is approximately 0.015 dB. As the frequency continues to increase, the response decreases again, approaching 0 dB near 181 MHz, and gradually falls to approximately −0.03 dB over the range from 190 MHz to 195 MHz. These results indicate that no obvious deep notches or abrupt weak-response points are present within the frequency band of interest. However, a certain degree of passband fluctuation and slow edge roll-off can still be observed. In terms of the phase response, the chain exhibits a smooth and monotonic variation over the entire analyzed frequency range. The phase curve is approximately linear and shows no evident phase discontinuities or severe bending.

### 3.3. Simulation Measurement-Chain Noise Construction

In the simulation analysis, the output signal of the measurement chain is generated according to the frequency-domain transmission model established in Section 3.2. For a given input signal spectrum X(f) and measurement-chain frequency response H(f), the noiseless output spectrum of the measurement chain is given by Equation (20). To simulate the observation noise introduced by the RF front-end, the acquisition chain, and unmodeled perturbations, Gaussian white noise is added to the noiseless measurement-chain output. The simulated signal can be expressed as:

(21) $$y\left(n\right)=y_{0}\left(n\right)+n\left(n\right)$$

The corresponding frequency-domain representation is given by:

(22) Y(f)=H(f)X(f)+N(f)

where n(n) denotes the additive noise sequence in the time domain, and N(f) denotes its Fourier transform. In this study, the noise is added at the output of the measurement chain. The noise power is determined according to the average power of the noiseless measurement-chain output signal and the preset signal-to-noise ratio. First, the average power of the noiseless output signal is calculated as follows:

(23) $$P_{y0}=\frac{1}{N}\sum_{n=0}^{N-1}{y_{0}}^{2}(n)$$

For a preset signal-to-noise ratio ${SNR}_{dB}$, the noise power is set as follows:

(24) $$P_{n}=\frac{P_{y_{0}}}{10^{{SNR}_{dB}/10}}$$

Therefore, the noise sequence can be expressed as:

(25) $$n\left(n\right)=\sqrt{P_{n}}w\left(n\right)$$

where w(n) is a real-valued Gaussian white noise sequence with zero mean and unit variance. The same noise construction method is adopted for all measurement-chain models to ensure consistent comparisons among different compensation methods.

## 4. Simulation Performance Analysis Under Different Measurement-Chain Conditions

To evaluate the recovery capability of different inverse compensation methods under various chain conditions, four representative chain scenarios were considered, including a smooth reference chain, a passband-edge attenuation chain, a pathological chain with multiple local fading notches, and a chain model based on measured S-parameters. For each scenario, simulations were performed for four inverse compensation methods, namely direct inverse compensation, Tikhonov-regularized inverse compensation, Wiener-type inverse compensation, and truncated inverse compensation.

**Figure 6:**
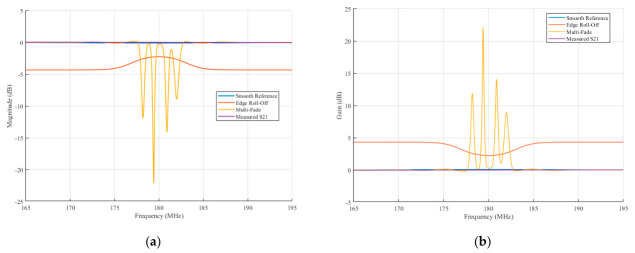
Comparison of chain magnitude responses and inverse compensation gains. (**a**) Magnitude responses of the four measurement-chain models. (**b**) Inverse compensation gains of the four measurement-chain models.

To further illustrate the distortion-correction mechanism of the compensation algorithm, Figure 6a presents the magnitude distortion characteristics of different chain models, and Figure 6b shows the corresponding compensation-gain distributions. It can be observed that the smooth reference chain and the measured S-parameter-based chain exhibit relatively small amplitude fluctuations over the analyzed frequency band, and their corresponding compensation gains remain close to 0 dB, indicating that only weak compensation is required for signal recovery in these cases. For the passband-edge attenuation chain, a continuous roll-off appears on both sides of the carrier frequency, and the corresponding compensation gain increases near the band edges, showing that the algorithm can inversely correct the attenuation in these regions. For the multiple local-fading chain, several deep local fading points are present near the carrier frequency, and the compensation gain increases significantly at the corresponding frequency locations, reaching its maximum at the deepest fading point. This indicates that the compensation-gain distribution can accurately follow the variation of weak-response regions in the chain. Overall, the compensation-gain curves show a clear correspondence with the magnitude-distortion distributions of the chain responses. Specifically, the more severe the chain attenuation is at a given frequency, the higher the compensation gain applied at that frequency. This result demonstrates that the proposed compensation method can perform targeted inverse correction according to different chain distortion characteristics, thereby verifying the effectiveness of the compensation strategy from a mechanistic perspective.

### 4.1. Analysis of Simulation Measurement-Chain Compensation Performance Under Fixed Parameter Settings

To ensure comparability among different compensation methods, the de-embedding compensation performance of the simulation measurement chains is first analyzed under fixed parameter settings. The simulated signal has a sampling rate of 1000 MHz, a carrier frequency of 180 MHz, and an effective signal bandwidth of 4 MHz. The detailed parameters are listed in Table 1. The measurement-chain models include the smooth reference chain, the passband-edge attenuation chain, the multiple local-fading ill-conditioned chain, and the chain model based on measured S-parameters.

For each measurement-chain model, the output signal is generated according to Y(f)=H(f)X(f)+N(f), where noise is added at the output of the measurement chain and the signal-to-noise ratio is set to 20 dB. Under this condition, the Tikhonov regularization parameter is set to $\lambda =10^{-5}$, and the truncation threshold for truncated de-embedding compensation is set to τ=0.08. The same observed signal is then compensated using the Direct, Tikhonov, Wiener, and Truncated methods, and the recovery performance of different methods is evaluated using NMSE and AmpError.

**Figure 7:**
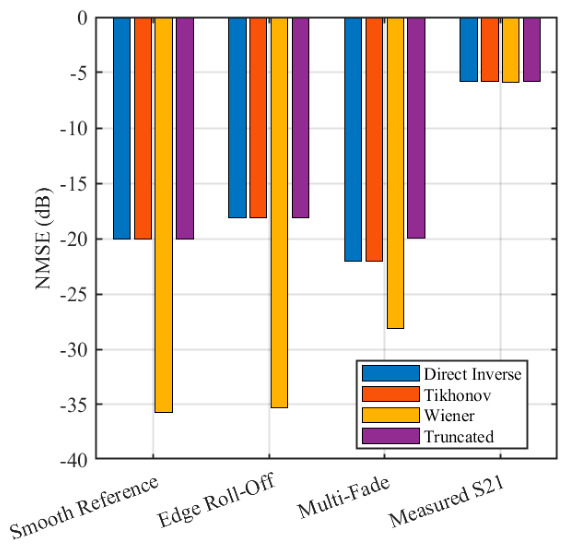
Comparison of NMSE Performance of Different Inverse Compensation Methods under Different Chain Models.

Table 2 and Figure 7 present the NMSE results of the four compensation methods under different measurement-chain models. As shown in the table, under the current fixed parameter settings, the NMSE results of the Direct, Tikhonov, and Truncated methods are generally close to each other. This indicates that the Tikhonov regularization term and the truncation threshold have relatively limited influence on the compensation results.

This can be explained by the fact that $\lambda =10^{-5}$ is small compared with $|H\left(f\right){|}^{2}$ at most frequency points, causing the Tikhonov compensation form to approximately degenerate into direct inverse compensation. Meanwhile, τ=0.08 activates the truncation constraint only at a few small-magnitude response points. Therefore, the compensation effect of the Truncated method is also close to that of the Direct method at most frequency points.

In contrast, the Wiener method achieves lower NMSE values under all measurement-chain conditions. For example, under the smooth reference chain and the passband-edge attenuation chain, its NMSE values are −35.718 dB and −35.311 dB, respectively, which are clearly better than those of the other three methods. This result indicates that the Wiener method can suppress noise amplification during compensation by introducing a constraint term related to the signal power spectrum and the noise power.

**Figure 8:**
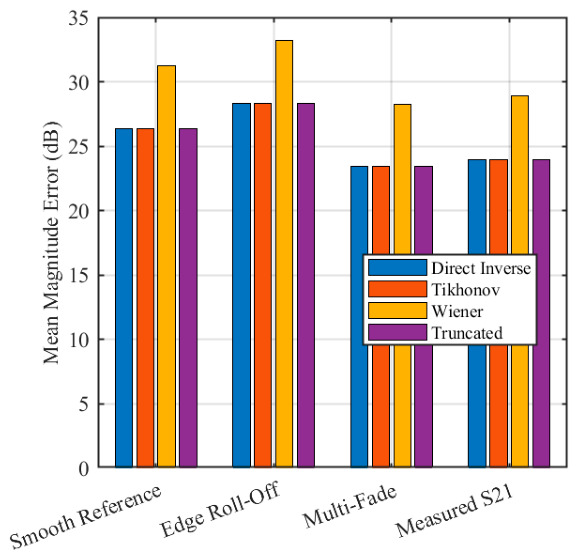
Comparison of AmpError Performance of Different Inverse Compensation Methods under Different Chain Models.

Table 3 and Figure 8 present the AmpError results of the four compensation methods. From the perspective of spectral-amplitude recovery, the Direct, Tikhonov, and Truncated methods produce almost identical results, indicating that their amplitude compensation performance differs only slightly under the current parameter settings.

Although the Wiener method achieves the best NMSE performance, its AmpError is relatively large. This suggests that, while the Wiener method reduces the overall reconstruction error, it imposes stronger suppression on some frequency components and therefore sacrifices a certain degree of spectral-amplitude fidelity.

Under the fixed parameter settings, the NMSE and AmpError results of the Direct, Tikhonov, and Truncated methods are relatively close. This phenomenon indicates that, when SNR = 20 dB and no extremely weak-response region appears in the measurement chain, the noise amplification problem associated with direct inverse compensation is not pronounced.

At the same time, $\lambda =10^{-5}$ is small compared with $|H\left(f\right){|}^{2}$ at most frequency points, causing the Tikhonov compensation form to approximately degenerate into direct inverse compensation. In addition, τ=0.08 activates the truncation constraint only at a few weak-response frequency points. Therefore, the Truncated method also remains close to the Direct method over most frequency points.

Compared with the artificially constructed measurement chains, the measurement-chain model based on measured $S_{21}$ exhibits stronger engineering non-idealities, resulting in relatively higher NMSE values. The smooth reference chain, passband-edge attenuation chain, and multiple local-fading chain are directly constructed using analytical models, so the measurement-chain response is strictly consistent with the simulated output. In contrast, the measured $S_{21}$ model is derived from VNA measurement data and may include practical amplitude fluctuations, phase delay, measurement noise, frequency interpolation errors, and reference-plane deviations.

Therefore, the higher NMSE observed for the measured $S_{21}$ model mainly reflects the non-ideal factors involved in practical measurement-chain response acquisition and mapping, rather than a failure of the compensation method itself. These results also indicate that experiments under fixed parameter settings are insufficient for fully evaluating the role of regularized methods. Further analysis should therefore be conducted through parameter sweeps and experiments under different SNR conditions.

### 4.2. Effects of Regularization Parameters and SNR Conditions on Compensation Performance

To investigate the influence of regularization parameter settings on compensation performance, single-parameter sweep experiments are conducted for lambda and tau. The influence of noise level on regularized inverse compensation is also considered by setting three representative SNR conditions, namely SNR = 0 dB, 10 dB, and 20 dB.

Specifically, SNR = 0 dB is used to simulate a strong-noise observation environment and to evaluate the ability of the regularized methods to suppress noise amplification caused by direct inverse compensation. SNR = 10 dB is used to characterize compensation stability under moderate noise conditions. SNR = 20 dB is used to analyze the gain variation of the regularized methods when direct inverse compensation already provides relatively good recovery performance.

In each experiment, only one parameter of the corresponding method is varied, while the other conditions remain unchanged. The compensation performance under different parameter settings is then evaluated using NMSE and AmpError.

#### 4.2.1. Effect of the Tikhonov Regularization Parameter λ on the Compensation Results

To analyze the effect of the Tikhonov regularization parameter on the de-embedding compensation results, a single-parameter sweep of λ is conducted while keeping the measurement-chain models and noise construction method unchanged. The results obtained by the Tikhonov method under different λ values are compared with those obtained by the Direct method. In the experiment, λ is varied from $10^{-8}$ to $5\times 10^{-1}$. The range from $10^{-8}$ to $10^{-2}$ is swept in increasing orders of magnitude, and larger values, including $5\times 10^{-2}$, $10^{-1}$, $2\times 10^{-1}$, and $5\times 10^{-1}$, are further included. All four measurement-chain models are tested, and the recovery performance is evaluated using NMSE and AmpError.

**Figure 9:**
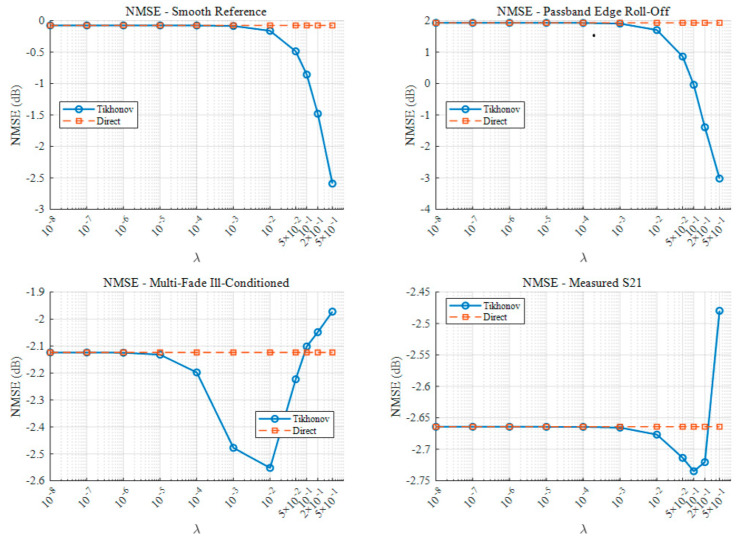
Effect of the Tikhonov Regularization Parameter on NMSE under SNR = 0 dB.

**Figure 10:**
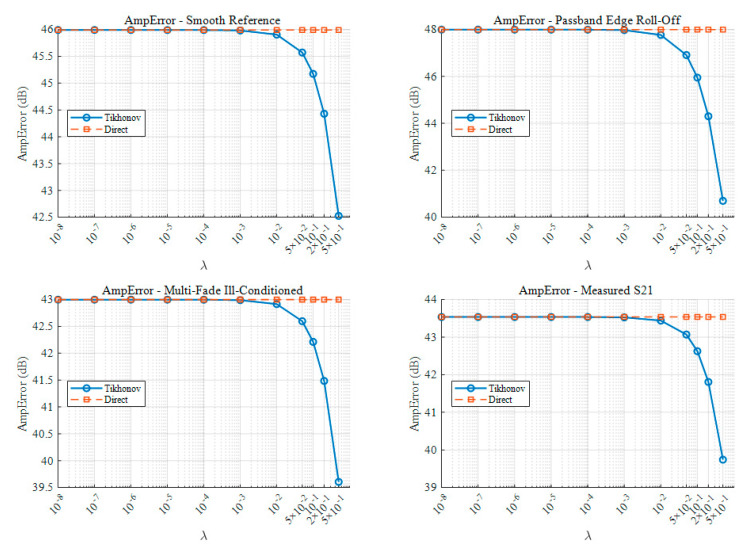
Effect of the Tikhonov Regularization Parameter on AmpError under SNR = 0 dB.

Under the experimental condition of SNR = 0 dB, the influence of λ on the compensation results of different measurement chains is evaluated. As shown in Figure 9 and Figure 10, when λ is small, especially in the range from $10^{-8}$ to $10^{-4}$, the Tikhonov curves almost overlap with the Direct baseline curves. This indicates that the regularization term is weak compared with $|H\left(f\right){|}^{2}$ in this case, and the compensation process approximately degenerates into direct inverse compensation. As a result, the suppression of noise amplification is not significant.

As λ gradually increases, both NMSE and AmpError of the Tikhonov method decrease for the smooth reference chain, the passband-edge attenuation chain, the multiple local-fading ill-conditioned chain, and the chain model based on measured S-parameters. For the smooth reference chain, when λ = 0.5, the NMSE decreases from −0.0737 dB for the Direct method to −2.5887 dB, and the AmpError decreases from 45.9908 dB to 42.5256 dB. The improvement is more pronounced for the passband-edge attenuation chain. When λ = 0.5, the NMSE decreases from 1.9342 dB to −3.0162 dB, and the AmpError decreases from 47.9973 dB to 40.6984 dB.

For the multiple local-fading ill-conditioned chain, the optimal NMSE is obtained near λ = 0.01, decreasing from −2.1237 dB to −2.5520 dB. However, when λ continues to increase, the NMSE deteriorates instead. This indicates that excessively strong regularization may weaken the recovery of effective signal components. For the measured $S_{21}$ chain, the improvement in NMSE achieved by the Tikhonov method is relatively limited, with a maximum improvement of only approximately 0.0706 dB. Nevertheless, AmpError still decreases to some extent as λ increases.

**Figure 11:**
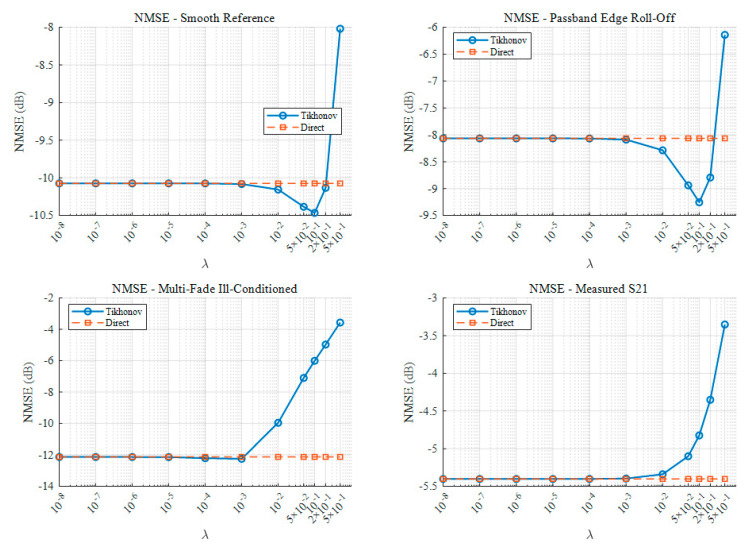
Effect of the Tikhonov Regularization Parameter on NMSE under SNR = 10 dB.

**Figure 12:**
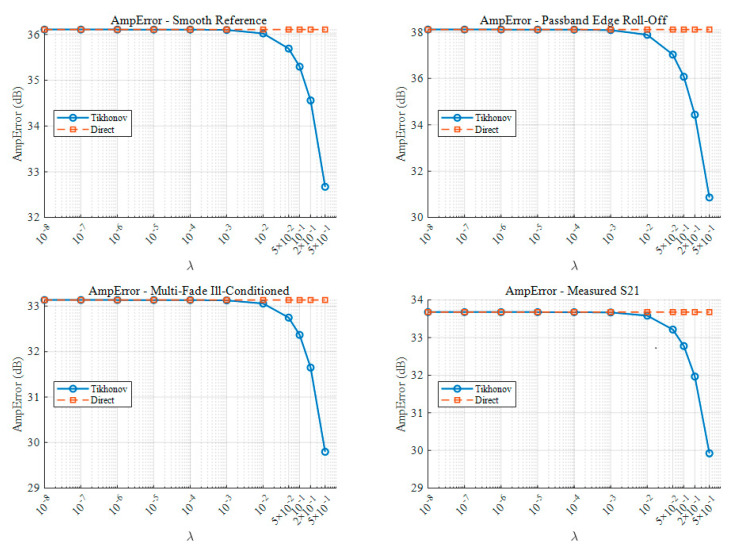
Effect of the Tikhonov Regularization Parameter on AmpError under SNR = 10 dB.

Under the SNR = 0 dB condition, Tikhonov regularization can improve the stability of direct inverse compensation to a certain extent. As shown in Figure 11 and Figure 12, this effect is particularly evident for the smooth reference chain and the passband-edge attenuation chain. However, for the complex fading chain and the measured S-parameter-based chain, an excessively large λ a may lead to under-compensation.

Under the experimental condition of SNR = 10 dB, the influence of the Tikhonov regularization parameter λ on the compensation results shows clear parameter dependence. When λ is in the range from $10^{-8}$ to $10^{-4}$, the NMSE and AmpError results of the Tikhonov method almost overlap with those of the Direct method. This indicates that the regularization constraint is weak in this range, and the compensation process is approximately equivalent to direct inverse compensation.

As λ increases, the NMSE of the smooth reference chain and the passband-edge attenuation chain is improved to some extent. In particular, for the passband-edge attenuation chain, the NMSE decreases from −8.0658 dB to −9.2547 dB when λ = 0.1, showing the most pronounced improvement. In contrast, the NMSE improvement is relatively limited for the multiple local-fading ill-conditioned chain and the measured $S_{21}$ chain. Moreover, obvious degradation occurs when λ becomes large, indicating that excessively strong regularization constraints may lead to under-compensation.

From the AmpError results, the amplitude error of all four measurement chains decreases as λ increases. This indicates that Tikhonov regularization can suppress abnormal amplitude amplification in direct inverse compensation. However, the reduction in AmpError does not necessarily correspond to a simultaneous improvement in NMSE. This is particularly evident in the multiple local-fading ill-conditioned chain and the measured $S_{21}$ chain, where a large λ reduces the amplitude error but significantly increases the overall recovery error.

Therefore, under the SNR = 10 dB condition, appropriate Tikhonov regularization can enhance the stability of the compensation process and plays a positive role in suppressing abnormal amplitude amplification and improving the recovery accuracy of some measurement chains.

**Figure 13:**
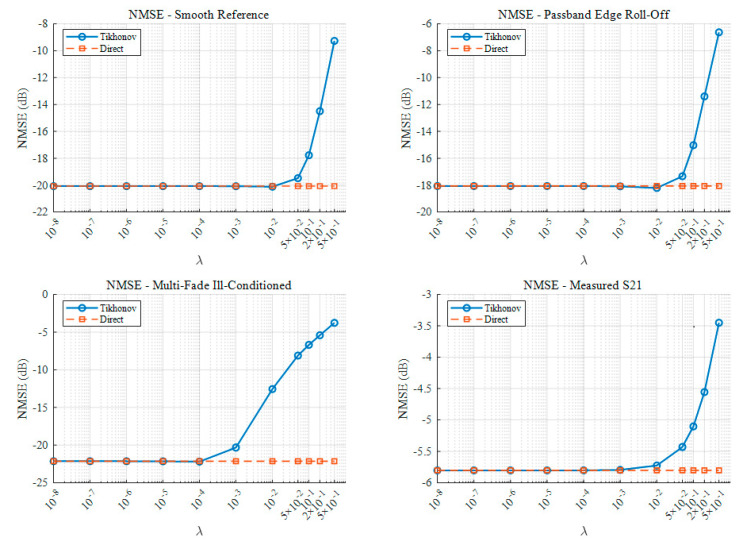
Effect of the Tikhonov Regularization Parameter on NMSE under SNR = 20 dB.

**Figure 14:**
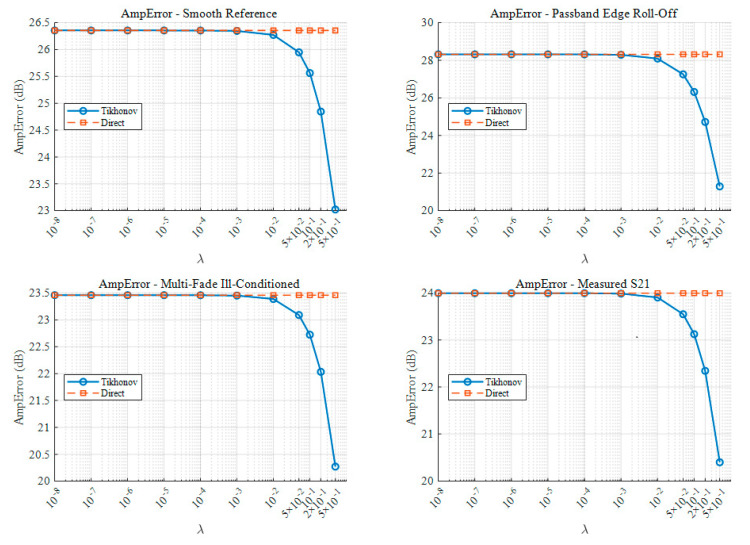
Effect of the Tikhonov Regularization Parameter on AmpError under SNR = 20 dB.

Under the SNR = 20 dB condition, the influence of the Tikhonov regularization parameter λ on the compensation results is weaker than that observed under the 0 dB and 10 dB conditions. As shown in Figure 13 and Figure 14, when λ is in the range from $10^{-8}$ to $10^{-4}$, the NMSE curve of the Tikhonov method almost overlaps with that of the Direct method. This indicates that, under this SNR condition, the noise amplification caused by direct inverse compensation has been partially alleviated, and weak regularization constraints have only a limited influence on recovery accuracy.

As λ increases, the NMSE of the smooth reference chain and the passband-edge attenuation chain shows a slight improvement. For the smooth reference chain, the NMSE decreases from −20.0737 dB for the Direct method to −20.1142 dB when λ = 0.01. For the passband-edge attenuation chain, the NMSE decreases from −18.0658 dB to −18.2113 dB when λ = 0.01. For the multiple local-fading ill-conditioned chain, the optimal result occurs at λ = $10^{-4}$, where the NMSE decreases from −22.1237 dB to −22.1757 dB. These results indicate that appropriate regularization can still provide a certain stabilizing effect during compensation.

From the AmpError results, all four measurement chains show a decreasing trend as λ increases, indicating that Tikhonov regularization can suppress abnormal amplitude amplification during inverse compensation. For example, when λ = 0.5, the AmpError of the passband-edge attenuation chain decreases from 28.3186 dB to 21.2920 dB, and that of the measured $S_{21}$ chain decreases from 23.9988 dB to 20.4039 dB.

However, although a larger λ can reduce the amplitude error, it may significantly deteriorate the NMSE. This effect is particularly evident in the multiple local-fading ill-conditioned chain, where the NMSE deteriorates to −3.7602 dB when λ = 0.5.

Under the SNR = 20 dB condition, Tikhonov regularization provides only limited improvement in NMSE. Nevertheless, with appropriate parameter settings, it can still enhance compensation stability and play a positive role in reducing amplitude error. During parameter selection, an excessively large λ should be avoided. Small or moderate regularization parameters are more suitable for moderately improving AmpError while keeping NMSE essentially stable. Based on the above results under SNR = 0 dB, 10 dB, and 20 dB, the influence of λ on Tikhonov regularized compensation can be summarized as follows.

Overall, the parameter-sweep results indicate that the effect of Tikhonov regularization is jointly determined by the value of λ, the noise level, and the response characteristics of the measurement chain. When λ is very small, especially in the range from 10^−8^ to 10^−4^, the regularization term is much weaker than |H(f)|^2^ at most frequency points, and the Tikhonov method behaves almost the same as direct inverse compensation. As λ increases, the inverse gain at weak-response frequency points is gradually constrained, which helps suppress abnormal amplitude amplification and improves compensation stability, particularly under low-SNR conditions and for chains with smooth or passband-edge attenuation responses. However, an excessively large λ may over-constrain the inverse compensation process and weaken the recovery of effective signal components, leading to under-compensation and NMSE degradation. Therefore, the Tikhonov regularization parameter should not be selected solely according to the reduction in AmpError. Instead, a trade-off between NMSE and AmpError should be considered. Under the tested conditions, small or moderate λ values are more suitable for maintaining overall reconstruction accuracy while improving the stability of de-embedding compensation.

#### 4.2.2. Effect of the Truncation Threshold τ on the Compensation Results

To analyze the effect of the truncation threshold τ on the compensation results of the truncated inverse compensation method, a single-parameter sweep of τ is conducted while keeping the measurement-chain models and noise construction method unchanged. The truncated inverse compensation results under different τ values are compared with those obtained by the Direct method.

Considering that the amplitude-frequency response levels differ among different measurement-chain models, the use of a fixed truncation threshold may lead to inconsistent truncation strengths in different scenarios. To improve the comparability of parameter settings, this study adopts a relative truncation threshold. Specifically, the minimum magnitude response within the effective signal bandwidth, $\min\;|H\left(f\right){|}_{band}$, is used as the reference, and the truncation threshold is set as $\tau =\alpha \min\;|H\left(f\right){|}_{band}$, where α is the relative threshold coefficient.

In the experiment, α is varied from 0.5 to 5. The specific values are 0.5, 0.8, 1.0, 1.05, 1.10, 1.20, 1.50, 2.00, 3.00, and 5.00. All four measurement-chain models are tested, and NMSE and AmpError are used to evaluate the variation in recovery accuracy and amplitude error under different truncation thresholds.

**Figure 15:**
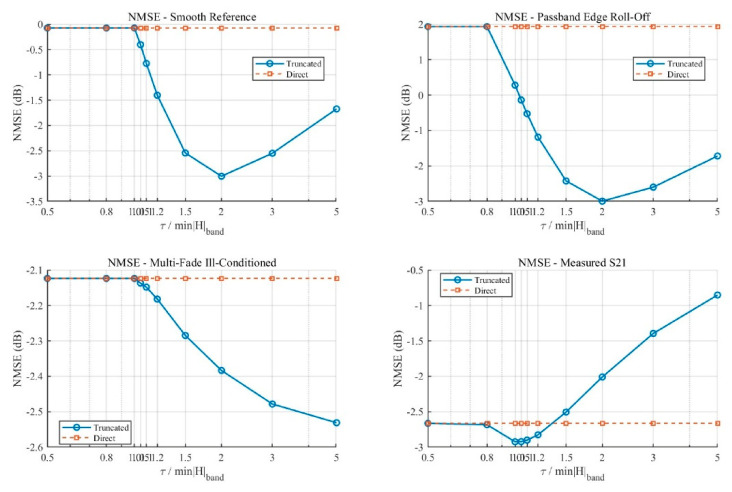
Effect of the Truncated Inverse Compensation Parameter on NMSE under SNR = 0 dB.

**Figure 16:**
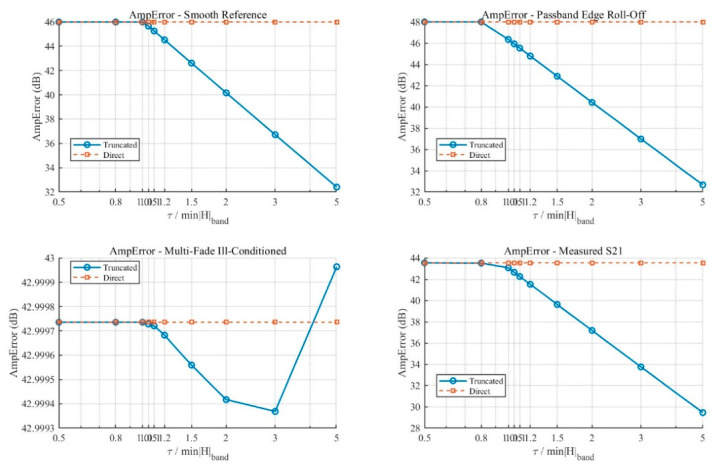
Effect of the Truncated Inverse Compensation Parameter on AmpError under SNR = 0 dB.

Under the SNR = 0 dB condition, the truncation threshold τ has a pronounced influence on the results of truncated inverse compensation. As shown in Figure 15 and Figure 16, when $\tau /{min|H|}_{band\;}$ is small, the results of the Truncated method are generally close to those of the Direct method, indicating that the truncation effect has not yet become significant. As the threshold increases, the excessive inverse compensation gain at weak-response frequency points is limited, leading to different degrees of improvement in both NMSE and AmpError.

From the NMSE results, the improvements are more pronounced for the smooth reference chain and the passband-edge attenuation chain. When $\tau /{min|H|}_{band\;}$ = 2.0, the NMSE of the smooth reference chain decreases from −0.0759 dB for the Direct method to −3.0086 dB, corresponding to an improvement of approximately 2.93 dB. For the passband-edge attenuation chain, the NMSE decreases from 1.9255 dB to −3.0051 dB, corresponding to an improvement of approximately 4.93 dB.The improvement for the multiple local-fading ill-conditioned chain is relatively limited. When $\tau /{min|H|}_{band\;}=\;$5.0, the NMSE decreases from −2.0236 dB to −2.4721 dB, corresponding to an improvement of approximately 0.45 dB. The measured $S_{21}$ chain is more sensitive to the threshold setting. A better result is obtained near $\tau /{min|H|}_{band\;}$ = 1.05, where the NMSE decreases from −2.6373 dB to −2.9010 dB. However, when the threshold continues to increase, the NMSE deteriorates noticeably, indicating that an excessively strong truncation constraint may lead to under-compensation.

From the AmpError results, as τ increases, the amplitude errors of the smooth reference chain, the passband-edge attenuation chain, and the measured $S_{21}$ chain decrease significantly. This indicates that the truncation threshold can effectively limit abnormal amplification in the magnitude spectrum. However, the continuous decrease in AmpError does not necessarily correspond to a continuous improvement in NMSE. Therefore, under the strong-noise condition of SNR = 0 dB, truncated inverse compensation provides a clear stabilizing effect and is suitable for suppressing noise amplification at weak-response frequency points.

**Figure 17:**
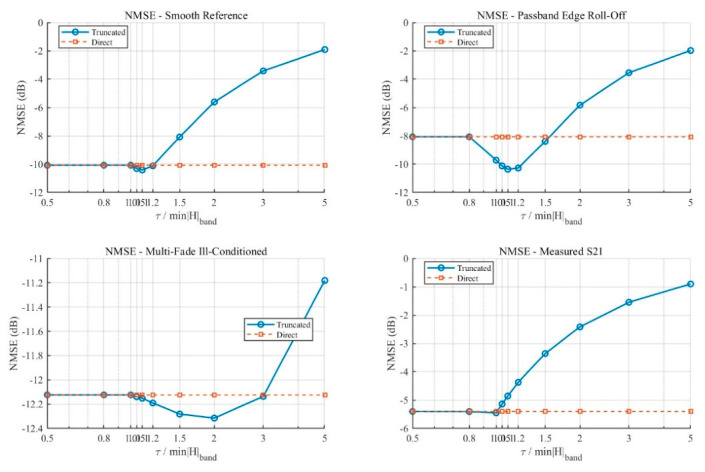
Effect of the Truncated Inverse Compensation Parameter on NMSE under SNR = 10 dB.

**Figure 18:**
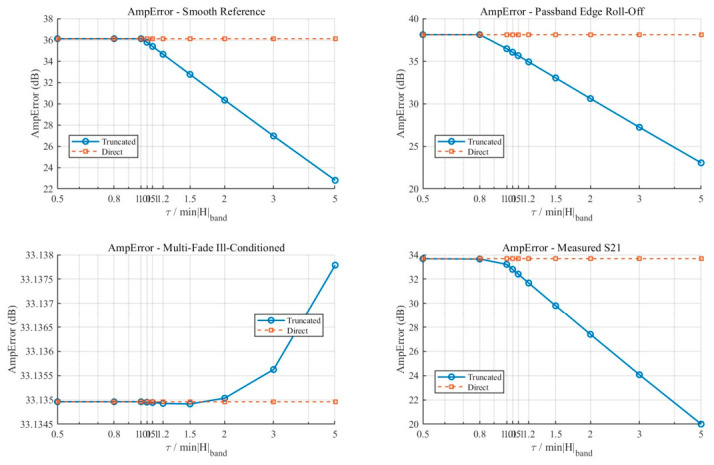
Effect of the Truncated Inverse Compensation Parameter on AmpError under SNR = 10 dB.

Under the SNR = 10 dB condition, the truncation threshold τ has a pronounced influence on the results of truncated inverse compensation. As shown in Figure 17 and Figure 18,when $\tau /{min|H|}_{band\;}$≤ 1.0, the results of the Truncated method are generally close to those of the Direct method, indicating that the truncation effect is still not significant. As the threshold increases appropriately, the compensation gain at weak-response frequency points is constrained, and the NMSE of some measurement chains is improved.

Among the tested chains, the passband-edge attenuation chain shows the most pronounced improvement. When $\tau /{min|H|}_{band\;}$= 1.10, the NMSE decreases from −8.0658 dB to −10.3512 dB, corresponding to an improvement of approximately 2.2854 dB. The smooth reference chain and the multiple local-fading ill-conditioned chain achieve improvements of approximately 0.3310 dB and 0.1909 dB, respectively.

From the AmpError results, as τ increases, the amplitude errors of the smooth reference chain, the passband-edge attenuation chain, and the measured $S_{21}$ chain all decrease significantly. For example, when $\tau /{min|H|}_{band\;}$= 5.00, the AmpError values of the passband-edge attenuation chain, the measured $S_{21}$ chain, and the smooth reference chain are improved by approximately 15.0603 dB, 13.6770 dB, and 13.3126 dB, respectively. This indicates that truncated inverse compensation has a strong ability to suppress abnormal amplitude amplification.

However, although a larger τ can reduce AmpError, it may also lead to NMSE degradation. Therefore, under the SNR = 10 dB condition, truncated inverse compensation can improve compensation stability, but the threshold should be selected by balancing recovery accuracy and amplitude error.

**Figure 19:**
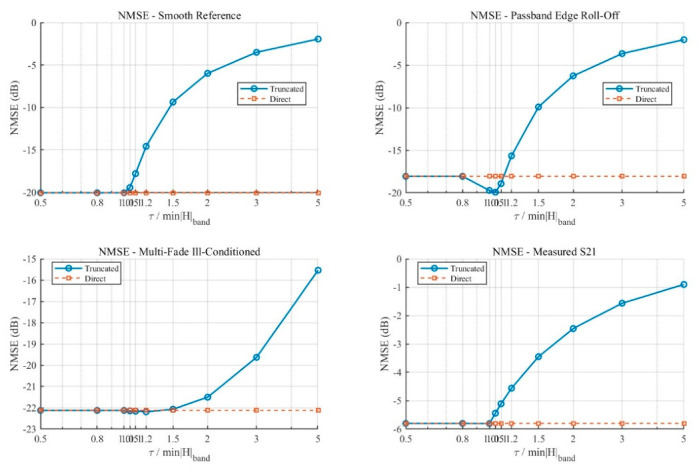
Effect of the Truncated Inverse Compensation Parameter on NMSE under SNR = 20 dB.

**Figure 20:**
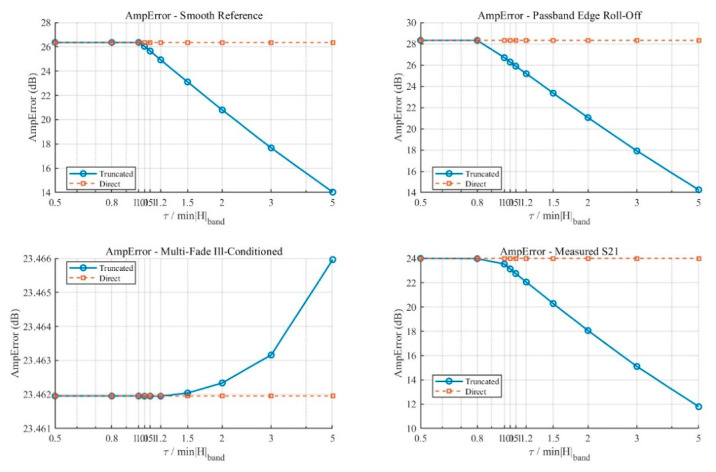
Effect of the Truncated Inverse Compensation Parameter on AmpError under SNR = 20 dB.

Under the SNR = 20 dB condition, the effect of truncated inverse compensation is weaker than that under low-SNR conditions. As shown in Figure 19 and Figure 20,when $\tau /{min|H|}_{band\;}$ ≤ 1.0, the results of the Truncated method are generally consistent with those of the Direct method. This indicates that the truncation condition is rarely activated in this range, and the compensation process is approximately equivalent to direct inverse compensation.

As the threshold increases, the truncation constraint begins to limit the compensation gain at weak-response frequency points. However, because the noise amplification problem has already been partially alleviated under the 20 dB condition, an excessively large truncation threshold is more likely to weaken the recovery of effective signal components, thereby leading to NMSE degradation.

From the NMSE results, the passband-edge attenuation chain shows the most pronounced improvement. When $\tau /{min|H|}_{band\;}$= 1.05, the NMSE decreases from −18.0658 dB for the Direct method to −19.9303 dB, corresponding to an improvement of approximately 1.8645 dB. For the multiple local-fading ill-conditioned chain, when $\tau /{min|H|}_{band\;}$ = 1.20, the NMSE decreases from −22.1237 dB to −22.1889 dB, corresponding to an improvement of approximately 0.0653 dB. The improvement for the measured $S_{21}$ chain is relatively small. When $\tau /{min|H|}_{band\;}$= 1.00, the NMSE only decreases from −5.8013 dB to −5.8065 dB.

From the AmpError results, as τ increases, the amplitude errors of the smooth reference chain, the passband-edge attenuation chain, and the measured $S_{21}$ chain all decrease noticeably. For example, when $\tau /{min|H|}_{band\;}$= 5.00, the AmpError of the passband-edge attenuation chain decreases from 28.3186 dB to 14.2441 dB, that of the smooth reference chain decreases from 26.3563 dB to 14.0243 dB, and that of the measured $S_{21}$ chain decreases from 23.9988 dB to 11.7870 dB. However, the corresponding NMSE values deteriorate significantly at this threshold. This indicates that simply increasing the truncation threshold can suppress abnormal amplitude amplification, but it may sacrifice overall recovery accuracy.

Therefore, under the SNR = 20 dB condition, truncated inverse compensation mainly acts to suppress amplitude error, whereas its improvement in NMSE is relatively limited. A small or moderate truncation threshold can improve recovery accuracy for some measurement chains, especially for the passband-edge attenuation chain. In contrast, an excessively large τ may lead to under-compensation. By combining the results under SNR = 0 dB, 10 dB, and 20 dB, the general influence of the truncation threshold can be further summarized as follows.

The parameter-sweep results indicate that the effectiveness of truncated inverse compensation is closely related to the relative truncation threshold $\tau /{min|H|}_{band\;}$, the SNR condition, and the response characteristics of the measurement chain. When $\tau /{min|H|}_{band\;}$ is small, the truncation constraint is rarely activated, and the Truncated method behaves almost the same as direct inverse compensation. As τ increases, the inverse compensation gain at weak-response frequency points is gradually limited, which effectively suppresses abnormal amplitude amplification and reduces AmpError. This stabilizing effect is more evident under low-SNR conditions, especially for the smooth reference chain and the passband-edge attenuation chain. However, a continuously increasing τ does not necessarily lead to better NMSE performance. When the truncation threshold is too large, the compensation of effective signal components may be weakened, resulting in under-compensation and degradation of overall recovery accuracy. This phenomenon becomes more pronounced under the SNR = 20 dB condition, where the noise amplification problem is less severe and excessive truncation may instead damage the recovered signal. Therefore, the truncation threshold should be selected by jointly considering NMSE and AmpError.

#### 4.2.3. Compensation Performance Analysis of Wiener-Type Inverse Filtering Under Different SNR Conditions

To analyze the compensation performance of Wiener-type inverse filtering under different noise levels, the power of the additive Gaussian white noise at the output of the measurement chain is varied while keeping the input signal, measurement-chain models, and compensation responses unchanged. The SNR is set to vary from −5 dB to 30 dB, and the results of Wiener-type inverse filtering are compared with those of the Direct method.

In the experiment, NMSE and AmpError are used as evaluation metrics to characterize the overall error of the recovered signal and the spectral-amplitude recovery deviation, respectively. These metrics are used to comprehensively evaluate the applicability of Wiener-type inverse filtering under different measurement-chain conditions and noise environments.

**Figure 21:**
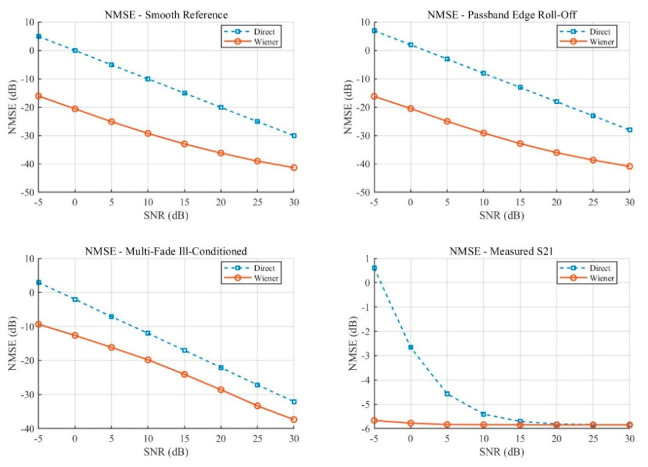
NMSE Comparison Results of Wiener-Type Inverse Filtering under Different SNR Conditions.

**Figure 22:**
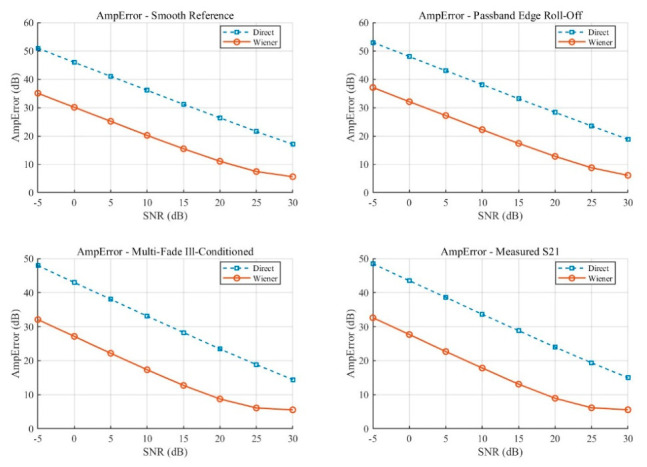
AmpError Comparison Results of Wiener-Type Inverse Filtering under Different SNR Conditions.

As shown in Figure 21 and Figure 22, Wiener-type inverse filtering achieves better compensation performance than the Direct method under different SNR conditions, and its advantage becomes more pronounced under low-SNR conditions.

The NMSE results show that, as the SNR increases from −5 dB to 30 dB, the NMSE values of both the Direct method and the Wiener method gradually decrease, indicating that the overall signal recovery error is reduced as the noise level decreases. Compared with the Direct method, the Wiener method achieves lower NMSE values under all SNR conditions.

Specifically, for the smooth reference chain, the Wiener method reduces the NMSE from −0.0792 dB for the Direct method to −20.5947 dB at SNR = 0 dB, corresponding to an improvement of approximately 20.5155 dB. At SNR = 20 dB, the NMSE is further reduced from −20.0755 dB to −36.1816 dB, corresponding to an improvement of approximately 16.1061 dB. For the passband-edge attenuation chain, the improvement is more pronounced. At SNR = 0 dB, the NMSE decreases from 1.9301 dB to −20.4798 dB, corresponding to an improvement of approximately 22.4099 dB. At SNR = 20 dB, the NMSE decreases from −18.0730 dB to −36.0255 dB, corresponding to an improvement of approximately 17.9525 dB.

For the multiple local-fading ill-conditioned chain, the Wiener method also maintains a stable advantage, achieving NMSE improvements of approximately 10.5955 dB and 6.5030 dB under the SNR = 0 dB and SNR = 20 dB conditions, respectively. For the measured $S_{21}$ chain, the NMSE improvement achieved by the Wiener method gradually decreases as the SNR increases. The improvement is approximately 3.1100 dB at SNR = 0 dB, whereas it is only approximately 0.0448 dB at SNR = 20 dB.

These results indicate that Wiener-type inverse filtering can effectively suppress noise amplification during inverse compensation, especially under low-SNR conditions. Its advantage is more significant for the smooth reference chain and the passband-edge attenuation chain, whereas the improvement is relatively limited for the measured $S_{21}$ chain at high SNR.

From the AmpError results, the Wiener method significantly reduces the amplitude error under all measurement-chain and SNR conditions. Taking SNR = 20 dB as an example, the AmpError of the smooth reference chain decreases from 26.3629 dB to 11.0495 dB, corresponding to an improvement of approximately 15.3133 dB. For the passband-edge attenuation chain, the AmpError decreases from 28.3136 dB to 12.7780 dB, corresponding to an improvement of approximately 15.5356 dB. For the multiple local-fading ill-conditioned chain, the AmpError decreases from 23.4788 dB to 8.7325 dB, corresponding to an improvement of approximately 14.7463 dB. For the measured $S_{21}$ chain, the AmpError decreases from 24.0177 dB to 8.9430 dB, corresponding to an improvement of approximately 15.0747 dB.

Overall, Wiener-type inverse filtering shows good adaptability to different noise levels. Under low-SNR conditions, it can significantly suppress the noise amplification caused by direct inverse compensation. Under medium- and high-SNR conditions, it can still maintain relatively low NMSE and AmpError. As the SNR increases, the recovery error of the Direct method itself gradually decreases, and the NMSE improvement of the Wiener method over the Direct method becomes smaller. Nevertheless, the Wiener method still maintains a clear advantage in suppressing amplitude error.

#### 4.2.4. Comprehensive Comparison of the Three Regularized Methods Under Different SNR Conditions

To further compare the applicability of Tikhonov regularization, Wiener-type inverse filtering, and truncated inverse compensation under different noise environments, the SNR is varied from −5 dB to 30 dB. The Direct method is used as a unified reference baseline, and the four compensation methods are comprehensively compared.

In the experiment, Wiener-type inverse filtering constructs the compensation weights according to the signal power spectrum and noise power. For Tikhonov regularization and truncated inverse compensation, parameter sweeps are performed under each SNR condition and for each measurement-chain model. The compensation results corresponding to the optimal parameters are then extracted according to the NMSE and AmpError metrics, as shown in Table 4.

According to the comprehensive comparison results of the four methods under different SNR conditions, clear performance differences can be observed among the methods. Overall, as the SNR increases from −5 dB to 30 dB, both NMSE and AmpError of the four methods show a decreasing trend, indicating that the compensation results are generally improved as the noise level decreases.

Among these methods, the Direct method serves as the baseline, and its performance mainly improves with the reduction in noise level. After selecting the NMSE-optimal parameters under each SNR condition, the overall results of Tikhonov regularization and truncated inverse compensation are relatively close to those of the Direct method, while certain improvements can still be observed for some measurement chains and low-SNR conditions. In contrast, Wiener-type inverse filtering achieves the best results under most measurement-chain and SNR conditions, demonstrating stronger noise suppression capability.

**Figure 23:**
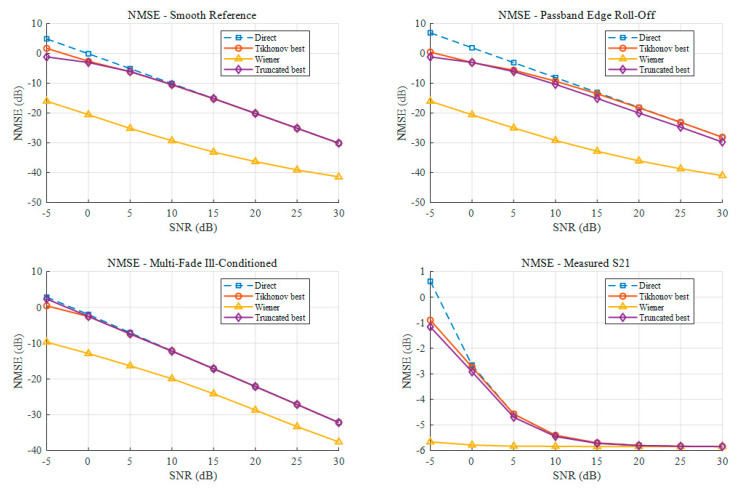
Comprehensive Comparison of NMSE Results of the Four Methods under Different SNR Conditions.

From the NMSE perspective, as shown in Figure 23. Wiener-type inverse filtering shows the most pronounced advantage in the smooth reference chain, the passband-edge attenuation chain, and the multiple local-fading ill-conditioned chain. For example, at SNR = 20 dB, the NMSE of the Wiener method in the smooth reference chain is −36.2587 dB, whereas those of the Direct method, the optimal Tikhonov method, and the optimal Truncated method are −20.0745 dB, −20.1127 dB, and −20.0745 dB, respectively. In the passband-edge attenuation chain, the Wiener method achieves an NMSE of −36.0083 dB, which is clearly better than those of the Direct method (−18.0792 dB), the optimal Tikhonov method (−18.2267 dB), and the optimal Truncated method (−19.9469 dB). In the multiple local-fading ill-conditioned chain, the Wiener method achieves an NMSE of −28.6990 dB, also outperforming the other three methods. For the measured $S_{21}$ chain, the NMSE differences among the methods are relatively small. Especially at high SNR, the NMSE values of the Direct, Tikhonov, and Truncated methods are already close to −5.8 dB, and the Wiener method provides only a slight improvement.

**Figure 24:**
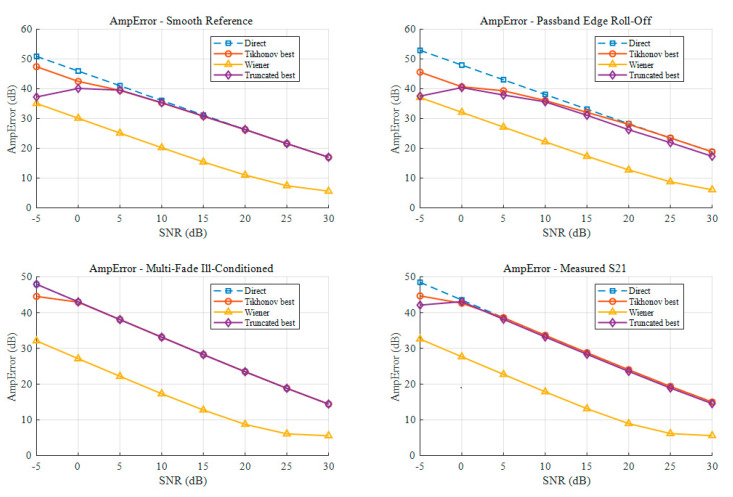
Comprehensive Comparison of AmpError Results of the Four Methods under Different SNR Conditions.

From the AmpError perspective, as shown in Figure 24. at SNR = 20 dB, the AmpError of the Wiener method in the smooth reference chain is 11.0500 dB, whereas those of the Direct method, the optimal Tikhonov method, and the optimal Truncated method are approximately 26.3579 dB, 26.2746 dB, and 26.3579 dB, respectively. In the passband-edge attenuation chain, the AmpError of the Wiener method is 12.7776 dB, which is markedly lower than those of the other methods. In the multiple local-fading ill-conditioned chain and the measured $S_{21}$ chain, the Wiener method also reduces AmpError to 8.7370 dB and 8.9476 dB, respectively. In contrast, the improvements in amplitude error achieved by Tikhonov regularization and truncated inverse compensation are relatively limited.

Overall, under the current simulation conditions, Wiener-type inverse filtering demonstrates better comprehensive compensation performance across different noise levels. Its advantages are particularly evident under low- and medium-SNR conditions, where it effectively suppresses noise amplification and reduces amplitude error. Tikhonov regularization and truncated inverse compensation can also improve the compensation results of the Direct method for certain measurement chains and specific SNR conditions. However, their performance gains are closely related to the regularization parameter, the truncation threshold, and the amplitude–frequency response characteristics of the measurement chain, indicating a certain degree of scenario dependence.

### 4.3. Summary

This chapter presents a simulation analysis of the de-embedding compensation performance of single-carrier signals under different measurement-chain conditions. Based on the smooth reference chain, the passband-edge attenuation chain, the multiple local-fading ill-conditioned chain, and the measured S-parameter-based chain model, the relationship between the magnitude response of the measurement chain and the inverse compensation gain is analyzed. The results show a clear inverse correspondence between the compensation gain and the magnitude response of the measurement chain. Specifically, stronger attenuation in the measurement chain requires a higher inverse compensation gain. In particular, for the multiple local-fading chain, local weak-response frequency points cause a significant increase in compensation gain, thereby increasing the risk of noise amplification and recovery instability.

Under fixed parameter settings, the Direct, Tikhonov, Wiener, and Truncated methods are compared. The results show that, when SNR = 20 dB and the regularization parameter and truncation threshold are relatively small, the NMSE and AmpError results of the Direct, Tikhonov, and Truncated methods are close to each other. This indicates that the regularization constraints have only a limited influence on the compensation results under these conditions. This result also suggests that the effectiveness of regularized methods is closely related to parameter settings, the degree of weak response in the measurement chain, and the noise level. Therefore, fixed-parameter experiments alone are insufficient to fully characterize their compensation performance.

Further parameter-sweep and SNR experiments show that Tikhonov regularization can suppress abnormal amplitude amplification with appropriate parameter settings and improve recovery accuracy for some measurement chains under low-SNR conditions. However, an excessively large λ may lead to under-compensation. The Truncated method improves stability by limiting the inverse compensation gain at weak-response frequency points and is particularly suitable for suppressing noise amplification under strong-noise conditions, although an excessively large threshold can also reduce overall recovery accuracy. Wiener-type inverse filtering constructs a frequency-dependent constraint using the signal power spectrum and noise power, and demonstrates better comprehensive compensation performance under the current simulation conditions. Its advantages are particularly evident under low- and medium-SNR conditions, where it provides clear improvements in NMSE and reductions in AmpError. Overall, this chapter verifies the stability limitation of direct inverse compensation in the presence of weak-response frequency points and noise perturbations, and demonstrates that regularized inverse compensation can improve the stability of single-carrier signal recovery by constraining the inverse compensation gain.

## 5. Experimental Validation Using Measured Signals

### 5.1. Experimental Platform and Measured Signal Acquisition

**Figure 25:**
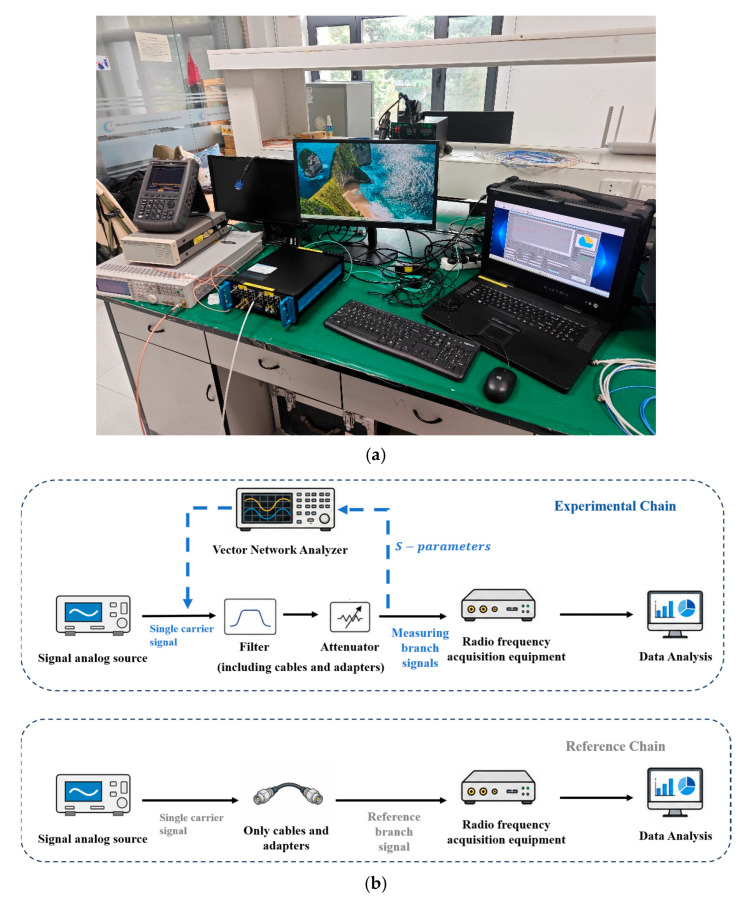
Experimental platform for verifying Measured signals: (**a**). Photograph of the Experimental Setup for Measured Single-Carrier Signal De-Embedding Compensation. (**b**). Schematic Diagram of the Measured Single-Carrier Signal De-Embedding Compensation Experiment.

To further verify the effectiveness of the frequency-domain inverse compensation methods for practically acquired signals, an experimental de-embedding compensation test was conducted using measured single-carrier signals, as shown in Figure 25. In the experiment, a Keysight N5182B vector signal generator (Keysight Technologies, Santa Rosa, CA, USA) was used to generate a QPSK-modulated single-carrier signal. The carrier frequency was set to 300 MHz, the symbol rate was 2 Msym/s, and Root Nyquist filtering was adopted for baseband pulse shaping.

The acquisition system directly sampled the RF signal at a sampling rate of 1000 MSps. The acquired data were then processed by digital down-conversion, low-pass filtering, and decimation to obtain the complex baseband signal. In the experiment, both the reference direct-connection signal and the observed signal passing through the filter-and-attenuator measurement chain were acquired. The $S_{21}$ measured by a VNA was used to construct the frequency response of the measurement chain. After being mapped onto the baseband frequency grid, this response was used for de-embedding compensation with the Direct, Tikhonov, Wiener, and Truncated methods.

### 5.2. Spectrum and Measurement-Chain Response Analysis of Measured Signals

**Figure 26:**
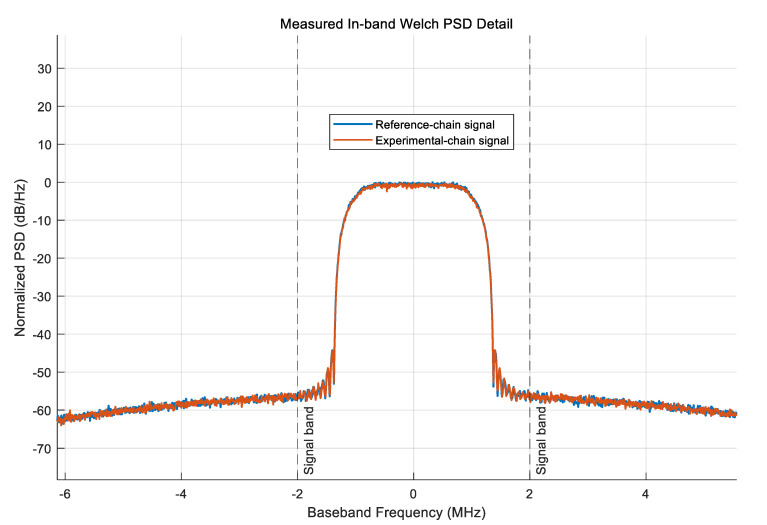
Spectrum of the Measured Single-Carrier Signal.

To verify the validity of the measured excitation signal, the spectrum of the acquired reference direct-connection signal is first analyzed. As shown in Figure 26, after down-conversion, the main energy of the QPSK-modulated single-carrier signal is concentrated around the baseband center and exhibits a continuous band-limited spectral distribution within the effective bandwidth. Owing to the Root Nyquist pulse shaping adopted by the signal generator, a certain roll-off transition appears at the spectral edges. The main signal energy is distributed within a range of several MHz around the carrier frequency, which is consistent with the spectral broadening characteristics of a modulated signal with a symbol rate of 2 Msym/s. No obvious abnormal spectral lines are observed in the out-of-band region, and the overall noise floor remains relatively stable. This indicates that the signal source output, RF acquisition, and down-conversion processing can provide a stable measured baseband signal.

The measurement-chain frequency response $\hat{H}(f)$ required for de-embedding compensation was obtained using a calibrated Agilent N5242A vector network analyzer (Keysight Technologies, Santa Rosa, CA, USA). In the experiment, the $S_{21}$ parameter of the experimental measurement chain was measured to obtain its magnitude attenuation and phase response. The measured response was then mapped onto the baseband frequency grid according to the carrier frequency, thereby constructing the measurement-chain response $\hat{H}(f)$ corresponding to the measured complex baseband signal.

**Figure 27:**
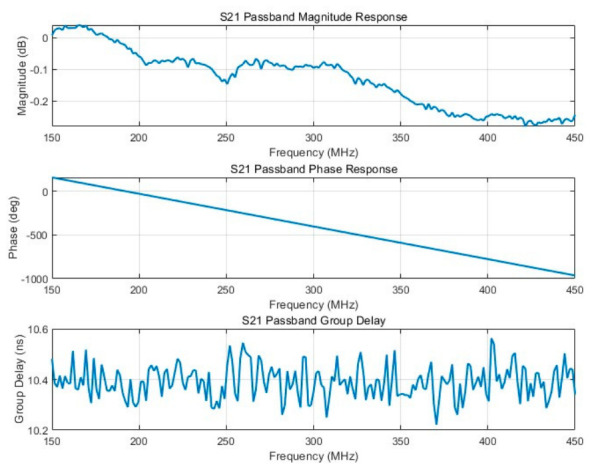
Experimental Measurement-Chain Frequency Response $\hat{H}(f)$.

As shown in Figure 27, within the frequency range of 150–450 MHz, the $S_{21}$ magnitude response of the experimental measurement chain is generally flat, with the magnitude variation mainly concentrated within the range of 0 to −0.25 dB. No obvious deep fading or severe amplitude distortion is observed. Overall, the measured chain exhibits relatively weak amplitude–frequency distortion around the carrier frequency, and can therefore be used as the basis for constructing $\hat{H}(f)$ in the subsequent frequency-domain inverse compensation using the Direct, Tikhonov, Wiener, and Truncated methods.

### 5.3. Analysis of De-Embedding Compensation Results for Measured Signals

Compared with the preceding simulation experiments, the four de-embedding compensation methods used for measured signal processing remain consistent in their basic compensation forms. All methods take the observed spectrum of the experimental measurement chain, $Y_{exp}\left(f\right)$ and the measurement-chain response $\hat{H}(f)$ mapped from $S_{21}$ as inputs. However, adaptations are made in terms of input signal acquisition, noise estimation, and parameter selection.

Under measured conditions, the true input signal X(f) at the input of the experimental measurement chain cannot be directly obtained. Therefore, this study uses the signal acquired from the reference measurement chain, $x_{ref,bb}\left(n\right)$, as a measured approximation of the input signal. Its spectrum, $X_{ref}\left(f\right)$, is obtained by FFT and is used to evaluate the compensation results.

Specifically, the Direct method is still used as the non-regularized baseline, and directly compensates the magnitude and phase of the observed signal passing through the experimental measurement chain using $\hat{H}(f)$. The Tikhonov method follows the regularized inverse compensation form used in the simulation. To avoid selecting an excessively large regularization parameter due to acquisition errors or very small numerical fluctuations, λ is swept from $10^{-8}$ to $5\times 10^{-1}$ in this study. The small-parameter range is swept in increasing orders of magnitude, while the larger-parameter range is used to observe the influence of overly strong regularization on spectral recovery.

The implementation of the Wiener method in the measured signal experiment differs most significantly from that in the simulation. In the simulation, $S_{x}\left(f\right)$ and $S_{n}\left(f\right)$ can be directly obtained from the known input signal and the preset noise. In contrast, under measured conditions, neither the true input signal nor the noise power spectrum can be directly determined. Therefore, the unknown true input signal is not substituted into the formula. Instead, the measured reference-chain signal is used to construct an estimate of the input power spectrum. Specifically, ${\hat{S}}_{x}\left(f\right)$ is calculated from $X_{ref}\left(f\right)$, as shown in Equation (26).

(26) $${\hat{S}}_{x}\left(f\right)=\frac{{|X}_{ref}\left(f\right){|}^{2}}{N}$$

where N denotes the number of FFT points. A moving average is then applied to reduce the fluctuation of the single-FFT spectral estimate. The noise power spectrum ${\hat{S}}_{n}\left(f\right)$ is estimated from the out-of-band region of the observed spectrum of the experimental measurement chain.

**Figure 28:**
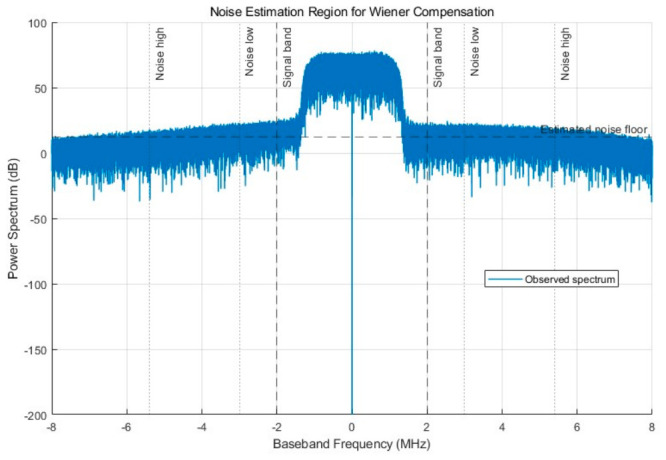
Baseband Spectral Distribution and Noise Floor Estimation of the Measured Signal.

According to the measured baseband spectral distribution shown in Figure 28, a guard band is set outside the effective signal bandwidth, and the region of 3 MHz < $|f_{bb}|$ < 5.4 MHz is selected. The median of the power spectrum in this region is calculated as the noise-floor estimate. This region avoids both the main lobe of the QPSK signal and the roll-off edge introduced by Root Nyquist filtering, while remaining within the effective passband of the low-pass filter used after down-conversion. Therefore, it can be used as a measured estimate of the noise constraint term in the Wiener method.

Consequently, in the measured signal processing, the Wiener method uses ${\hat{S}}_{x}\left(f\right)$ and ${\hat{S}}_{n}\left(f\right)$, rather than an assumed known true input power spectrum.

The Truncated method also follows the truncated inverse compensation principle used in the simulation. However, in the measured signal processing, the minimum value of $|\hat{H}(f)|$ within the effective signal bandwidth is not directly used as the truncation threshold. This is because the measured $S_{21}$ may be affected by measurement fluctuations, interpolation errors, and local abnormal points, and directly using the minimum value may lead to unstable threshold settings.

Therefore, this study uses a low percentile of ∣H(f)∣ within the effective signal bandwidth as a robust weak-response reference. Specifically, the 5th percentile, $H_{low}$, is selected, and the truncation threshold is set as $\tau =c_{\tau }H_{low}$. The parameter $c_{\tau }$ is swept from 0.5 to 3.0. Meanwhile, the in-band activation ratio satisfying $|\hat{H}(f)|\;<\tau$ is calculated, and parameter values with an activation ratio not exceeding 10% are preferentially selected to avoid full-band under-compensation caused by an excessively strong truncation constraint.

Table 5 presents the comparison results of different metrics before and after compensation for the measured signal. As shown in the table, the NMSE before compensation is −18.7808 dB. After applying the Direct method, the NMSE decreases to −37.9458 dB, corresponding to an improvement of approximately 19.1650 dB. This indicates that constructing the measurement-chain frequency response using the measured $S_{21}$ and performing frequency-domain de-embedding compensation can effectively improve the consistency between the signal passing through the experimental measurement chain and the reference-chain signal. This result verifies the feasibility of the frequency-domain inverse compensation model for practically acquired data.

From the comparison among different compensation methods, the Direct, Tikhonov, and Truncated methods show almost identical NMSE and AmpError results. Specifically, the NMSE values of the Direct and Tikhonov methods are both −37.9458 dB, while that of the Truncated method is −37.9459 dB. The differences among the three methods are negligible. Their AmpError values are all approximately 4.4613 dB. This phenomenon is consistent with the simulation results obtained for the smooth reference chain and the measured S-parameter-based chain model. When the magnitude response of the measurement chain is relatively flat within the effective signal bandwidth and no obvious local weak-response region appears, direct inverse compensation does not cause significant noise amplification. In this case, the additional effects of the Tikhonov regularization term and the truncation constraint are relatively limited, and their compensation forms become close to that of the Direct method.

The NMSE of the Wiener method is −38.2321 dB, which is further reduced by approximately 0.2863 dB compared with the Direct method. This indicates that the Wiener method can still improve the overall mean square error by introducing constraints related to the input power spectrum and noise power spectrum. However, its AmpError increases to 4.5431 dB, which is slightly higher than those of the other three compensation methods. This suggests that, while the Wiener method improves the overall recovery error, it applies stronger suppression to some frequency components and may therefore introduce a certain loss of spectral-amplitude fidelity. This observation is also consistent with the simulation results.

Overall, the measured results in Table 5 show that frequency-domain de-embedding compensation based on measured $S_{21}$ can significantly improve the overall recovery accuracy of the signal passing through the experimental measurement chain. Meanwhile, the performance of different regularized methods is clearly affected by the response characteristics of the measurement chain. Under the current measured condition, where the magnitude response of the measurement chain is relatively flat, the Tikhonov and Truncated methods provide no obvious improvement over the Direct method, whereas the Wiener method achieves slightly better NMSE performance.

**Figure 29:**
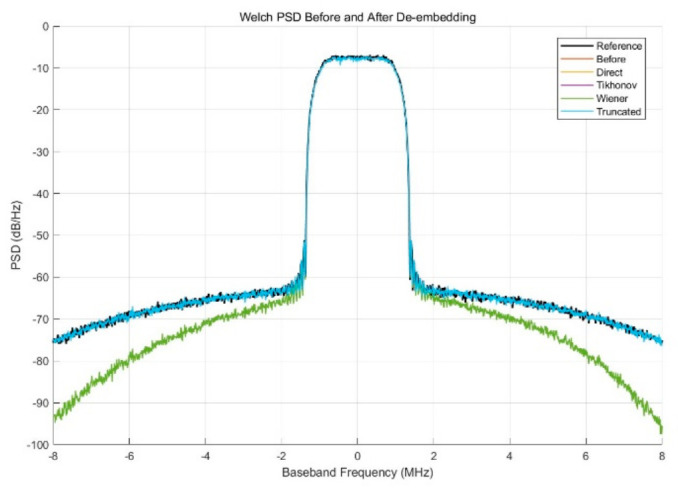
Comparison of Welch PSD before and after Compensation.

As shown in Figure 29, the comparison of Welch PSD before and after compensation shows that the Reference, Before, Direct, Tikhonov, and Truncated results almost overlap within the main signal bandwidth. The Wiener method exhibits more pronounced noise suppression in the out-of-band region. Its NMSE is −38.2321 dB, which is slightly better than that of the Direct method. However, its in-band amplitude spectral error and Welch PSD error increase to 4.5431 dB and 0.7241 dB, respectively.

This indicates that the Wiener method reduces the compensation gain of some frequency components by introducing a noise-power constraint. As a result, it can improve the overall mean square error, but may also sacrifice a certain degree of spectral-amplitude fidelity.

The parameter-sweep results in Table 6 further verify the above observations. For the Tikhonov method, when λ is small, the result is almost identical to that of the Direct method. As λ increases, the NMSE shows only a slight improvement, whereas AmpError increase noticeably. This indicates that overly strong regularization may lead to insufficient spectral compensation.

For the Truncated method, when $c_{\tau }\leq 0.8$, the truncation constraint is not activated, and the result degenerates into that of the Direct method. When $c_{\tau }=1.0$, only approximately 5% of the in-band frequency points activate the truncation constraint, and the result remains close to that of the Direct method. When $c_{\tau }\geq 1.1$, the truncation ratio reaches 100%. Although the NMSE changes only slightly, the amplitude spectral error and PSD error increase significantly, indicating that full-band truncation causes under-compensation.

Overall, the measured results are consistent with the conclusions obtained from the simulation analysis. When the measurement-chain response is relatively flat and no obvious weak-response frequency points are present, regularized inverse compensation provides no clear advantage over direct inverse compensation. Its main benefit is more likely to appear under low-SNR conditions or in measurement chains with local weak-response regions.

### 5.4. Summary

This chapter presents an experimental validation of frequency-domain de-embedding compensation using measured single-carrier signals. In the experiment, signals were acquired through both the reference measurement chain and the experimental measurement chain. The $S_{21}$ parameter measured by a calibrated vector network analyzer was then used to construct the measurement-chain response $\hat{H}(f)$ corresponding to the measured complex baseband signal. On this basis, the four frequency-domain inverse compensation methods established in the preceding sections, namely the Direct, Tikhonov, Wiener, and Truncated methods, were applied to measured signal processing, thereby extending the simulation-based model to practically acquired data.

The measured results show that $S_{21}$-based frequency-domain de-embedding compensation can effectively improve the consistency between the signal passing through the experimental measurement chain and the reference-chain signal. The NMSE before compensation is −18.7808 dB, and decreases to −37.9458 dB after compensation using the Direct method. This demonstrates that measurement-chain response mapping and frequency-domain inverse compensation can effectively correct the amplitude and phase effects introduced by the measured chain. To address the difficulty of directly obtaining the true input signal and noise power spectrum under measured conditions, the reference-chain signal is used to estimate the input power spectrum for the Wiener method, while the out-of-band region of the experimental-chain signal is used to estimate the noise power spectrum. This improves the operability of parameter setting for measured data.

The comparison among different methods shows that the Tikhonov and Truncated methods produce results close to those of the Direct method, whereas the Wiener method provides a slight improvement in NMSE. This observation is consistent with the simulation results. When the measured chain response is relatively flat within the effective bandwidth and no obvious weak-response frequency points are present, direct inverse compensation can already achieve satisfactory performance, and the additional contribution of regularization is limited. In contrast, under low-SNR conditions, in the presence of local weak-response regions, or when direct inverse compensation may introduce noise amplification, regularized inverse compensation methods can better demonstrate their stabilizing advantages. Overall, the measured experiment verifies the engineering feasibility of the proposed frequency-domain de-embedding compensation framework and further clarifies the applicability boundaries of different inverse compensation methods.

## 6. Conclusions

This paper investigated direct inverse and regularized inverse de-embedding compensation methods to address the degradation of recovery accuracy caused by amplitude fluctuation, phase distortion, and noise amplification in single-carrier signal measurement chains. A frequency-domain de-embedding model for single-carrier signals was established based on a linear time-invariant system. The analysis showed that, when weak-response frequency points or strong observation noise are present in the measurement chain, direct inverse compensation may produce excessive inverse gain and noise amplification, making the de-embedding process behave as an ill-posed inverse problem. To address this issue, three regularized inverse compensation methods, namely Tikhonov, Wiener, and Truncated methods, were introduced on the basis of the Direct method, and a unified frequency-domain compensation framework was constructed.

In the simulation analysis, a narrowband single-carrier signal was generated, and four measurement-chain models were designed, including a smooth reference chain, a passband-edge attenuation chain, a multiple local-fading ill-conditioned chain, and a measured S-parameter-based chain model. The results showed that the magnitude-response characteristics of the measurement chain directly determine the compensation-gain distribution. A weaker chain response corresponds to a higher inverse compensation gain. In the fixed-parameter experiments, the Direct, Tikhonov, and Truncated methods produced relatively similar results for most measurement chains, whereas the Wiener method achieved better NMSE performance. This indicates that the Wiener method can improve overall recovery stability by introducing constraints related to the signal power spectrum and noise power spectrum. Further parameter-sweep and SNR experiments demonstrated that the effectiveness of regularized methods is closely related to the ill-conditioning degree of the measurement chain, the noise level, and the parameter settings. When the regularization or truncation parameter is too small, the method tends to degenerate into direct inverse compensation. In contrast, an excessively large parameter may lead to under-compensation of effective signal components. Therefore, regularized inverse compensation is more suitable for low-SNR conditions, local weak-response regions, or scenarios where direct inverse compensation may introduce significant noise amplification.

In the measured-signal validation, a single-carrier signal acquisition platform consisting of a reference measurement chain and an experimental measurement chain was established. The $S_{21}$ parameter measured by a calibrated vector network analyzer was used to construct the measured chain response $\hat{H}(f)$. The experimental results showed that frequency-domain de-embedding compensation based on the measured $S_{21}$ can effectively improve the consistency between the signal passing through the experimental measurement chain and the reference-chain signal. The NMSE before compensation was −18.7808 dB, and it decreased to −37.9458 dB after compensation using the Direct method. To address the difficulty of directly obtaining the true input signal and the noise power spectrum under measured conditions, the reference-chain signal was used to estimate the input power spectrum for the Wiener method, while the out-of-band region of the experimental-chain signal was used to estimate the noise power spectrum. This improved the feasibility of the method for measured data processing.

The combined simulation and measured results indicate that the proposed frequency-domain de-embedding compensation framework can effectively describe the transmission and recovery process of single-carrier signals in measurement chains, and can be used to compare the applicability of different inverse compensation methods. The measured results further confirm the simulation conclusions. When the measurement-chain response is relatively flat and no obvious weak-response frequency points are present, the Direct method can already achieve satisfactory compensation performance, and the additional effects of the Tikhonov and Truncated methods are limited. The Wiener method shows a certain advantage in terms of NMSE, but may sacrifice part of the spectral-amplitude fidelity. Overall, the main value of regularized inverse compensation lies in scenarios where weak-response regions or high noise-amplification risks are present in the measurement chain. This study provides a modeling basis, method comparison, and measured-signal validation for frequency-domain de-embedding compensation in single-carrier signal measurement chains.

Future work can further extend the proposed framework to modulation-quality preservation and active distortion correction in complex chain environments. This study mainly focused on post-compensation at the receiver side for single-carrier signal recovery in measurement chains. On this basis, future research can extend the objective from frequency-domain recovery alone to modulation-quality metrics, such as spectral shape, error vector magnitude, and constellation distribution. In practical testing and transmission chains, modulated signals may be affected simultaneously by strong frequency-selective fading, low SNR, nonlinear amplitude and phase distortion, and measurement or estimation errors in H(f). These non-ideal factors may further intensify noise amplification and model mismatch during inverse compensation. Therefore, future work can further investigate robust de-embedding methods for harsh chain environments by incorporating chain-response errors, local weak-response regions, and observation noise into a unified regularized inverse compensation model. In addition, the frequency-domain compensation concept proposed in this paper can be combined with predistortion techniques for modulated signals. The measured or estimated chain response $\hat{H}(f)$, together with its uncertainty, can be used to construct a frequency-domain pre-correction model, so that amplitude and phase distortions can be pre-compensated before the signal enters the measurement chain or transmission chain. The receiver-side de-embedding results can then be used to optimize the predistortion parameters, forming a closed-loop processing framework that integrates chain modeling, predistortion correction, measured feedback, and de-embedding evaluation. This would extend the proposed method from receiver-side signal recovery to modulation-quality control and active chain-distortion correction, thereby supporting high-fidelity modulated-signal generation and evaluation in complex measurement chains and practical transmission chains.

## Figures and Tables

**Table 1:** Parameters of the Simulated Signal.

Parameter	Symbol	Value
Sampling rate	$f_{s}$	1000 MHz
Number of samples	N	$2^{16}$
Carrier frequency	$f_{c}$	180 MHz
Effective signal bandwidth	B	4 MHz

**Table 2:** Comparison of the NMSE(dB) Results of the Four Compensation Methods.

Scenario	Direct	Tikhonov	Wiener	Truncated
Smooth Reference Chain	−20.0750	−20.0750	−35.7180	−20.0750
Passband Edge Roll-Off Chain	−18.0910	−18.0920	−35.3110	−18.0910
Multi-Fade Ill-Conditioned Chain	−22.032	−22.027	−28.126	−19.9800
Measured S-Parameter-Based Chain	−5.8314	−5.8313	−5.8761	−5.8314

**Table 3:** Comparison of the AmpError (dB) Results of the Four Compensation Methods.

Scenario	Direct	Tikhonov	Wiener	Truncated
Smooth Reference Chain	26.3330	26.3330	31.2130	26.3330
Passband Edge Roll-Off Chain	28.2990	28.2990	33.1860	28.2990
Multi-Fade Ill-Conditioned Chain	23.4490	23.4490	28.2830	23.4500
Measured S-Parameter-Based Chain	23.9600	23.9600	28.9090	23.9600

**Table 4:** Optimal Values of the Tikhonov Regularization Parameter and the Relative Threshold Coefficient for Truncated Inverse Compensation under Different SNR Conditions.

Scenario	Parameter	0 dB	10 dB	20 dB	30 dB
Smooth Reference	$\lambda_{opt}$	0.5	0.1	0.01	0.001
	$\alpha_{opt}$	2.0	1.1	0.5	0.5
Passband Edge Roll-Off	$\lambda_{opt}$	0.5	0.1	0.01	0.001
	$\alpha_{opt}$	2.0	1.1	1.05	1.0
Multi-Fade Ill-Conditioned	$\lambda_{opt}$	0.01	0.001	$10^{-4}$	$10^{-4}$
	$\alpha_{opt}$	5.0	2.0	0.5	1.05
Measured $S_{21}$	$\lambda_{opt}$	0.1	$10^{-8}$	$10^{-8}$	$10^{-8}$
	$\alpha_{opt}$	1.0	1.0	1.0	1.0

The Direct method does not involve any regularization parameter, and Wiener-type inverse filtering does not require parameter sweeping in this experiment. Therefore, only the optimal Tikhonov regularization parameter $\;\lambda_{opt}$ and the optimal relative threshold coefficient α_opt for truncated inverse compensation are listed in the table, where $\alpha_{opt}$ = $\tau_{opt}$/min |${H|}_{band}$.

**Table 5:** Comparison of the Four De-Embedding Methods.

Method	NMSE/dB	AmpError/dB
Before	−18.7808	4.4608
Direct	−37.9458	4.4613
Tikhonov	−37.9458	4.4613
Wiener	−38.2321	4.5431
Truncated	−37.9459	4.4613

**Table 6:** Typical Parameter Settings of Regularized Methods for the Measured Signal.

Method	Parameter Setting	NMSE/dB	AmpError/dB	Interpretation
Tikhonov	$\lambda =10^{-5}$	−37.9458	4.4613	Close to Direct
Tikhonov	$\lambda =10^{-1}$	−37.9464	4.7494	Increased spectral error
Tikhonov	$\lambda =5\times 10^{-1}$	−37.9506	5.8211	Over-regularization
Truncated	$c_{\tau }$ = 0.8	−37.9458	4.4613	Not activated
Truncated	$c_{\tau }$ = 1.0	−37.9459	4.4613	Slightly activated
Truncated	$c_{\tau }$ = 1.5	−37.9527	5.8181	Full-band truncation

## Data Availability

The original contributions presented in this study are included in the article. Further inquiries can be directed to the corresponding author.

## Abbreviations

The following abbreviations are used in this manuscript:

NMSE	Normalized Mean Square Error
AmpError	Amplitude Error
